# Synthesis and Anticancer Evaluation of 4-Chloro-2-((5-aryl-1,3,4-oxadiazol-2-yl)amino)phenol Analogues: An Insight into Experimental and Theoretical Studies

**DOI:** 10.3390/molecules28166086

**Published:** 2023-08-16

**Authors:** Obaid Afzal, Amena Ali, Abuzer Ali, Abdulmalik Saleh Alfawaz Altamimi, Manal A. Alossaimi, Md Afroz Bakht, Salahuddin, Mubarak A. Alamri, Md. Faiyaz Ahsan, Mohamed Jawed Ahsan

**Affiliations:** 1 Department of Pharmaceutical Chemistry, College of Pharmacy, Prince Sattam Bin Abdulaziz University, Al-Kharj 11942, Saudi Arabia; 2 Department of Pharmaceutical Chemistry, College of Pharmacy, Taif University, P.O. Box 11099, Taif 21944, Saudi Arabia; 3 Department of Pharmacognosy, College of Pharmacy, Taif University, P.O. Box 11099, Taif 21944, Saudi Arabia; 4 Department of Chemistry, College of Science and Humanity Studies, Prince Sattam Bin Abdulaziz University, Al-Kharj 11942, Saudi Arabia; 5 Department of Pharmaceutical Chemistry, Noida Institute of Engineering and Technology, (Pharmacy Institute), Knowledge Park-2, Greater Noida 201 306, India; 6 Department of Chemistry, Bihar National College, Patna 800 004, India; 7 Department of Pharmaceutical Chemistry, Maharishi Arvind College of Pharmacy, Jaipur 302 039, India

**Keywords:** anticancer, antibacterial, DNA gyrase, molecular docking, 1,3,4-oxadiazole, tubulin

## Abstract

We report herein the synthesis, docking studies and biological evaluation of a series of new 4-chloro-2-((5-aryl-1,3,4-oxadiazol-2-yl)amino)phenol analogues (**6a-h**). The new compounds were designed based on the oxadiazole-linked aryl core of tubulin inhibitors of IMC-038525 and IMC-094332, prepared in five steps and further characterized via spectral analyses. The anticancer activity of the compounds was assessed against several cancer cell lines belonging to nine different panels as per National Cancer Institute (NCI US) protocol. 4-Chloro-2-((5-(3,4,5-trimethoxyphenyl)-1,3,4-oxadiazol-2-yl)amino)phenol (**6h**) demonstrated significant anticancer activity against SNB-19 (PGI = 65.12), NCI-H460 (PGI = 55.61), and SNB-75 (PGI = 54.68) at 10 µM. The compounds were subjected to molecular docking studies against the active site of the tubulin–combretastatin A4 complex (PDB ID: 5LYJ); they displayed efficient binding and ligand **4h** (with docking score = −8.030 kcal/mol) lay within the hydrophobic cavity surrounded by important residues Leu252, Ala250, Leu248, Leu242, Cys241, Val238, Ile318, Ala317, and Ala316. Furthermore, the antibacterial activity of some of the compounds was found to be promising. 4-Chloro-2-((5-(4-nitrophenyl)-1,3,4-oxadiazol-2-yl)amino)phenol (**6c**) displayed the most promising antibacterial activity against both Gram-negative as well as Gram-positive bacteria with MICs of 8 µg/mL and a zone of inhibition ranging from 17.0 ± 0.40 to 17.0 ± 0.15 mm at 200 µg/mL; however, the standard drug ciprofloxacin exhibited antibacterial activity with MIC values of 4 µg/mL.

## 1. Introduction

**Figure 1:**
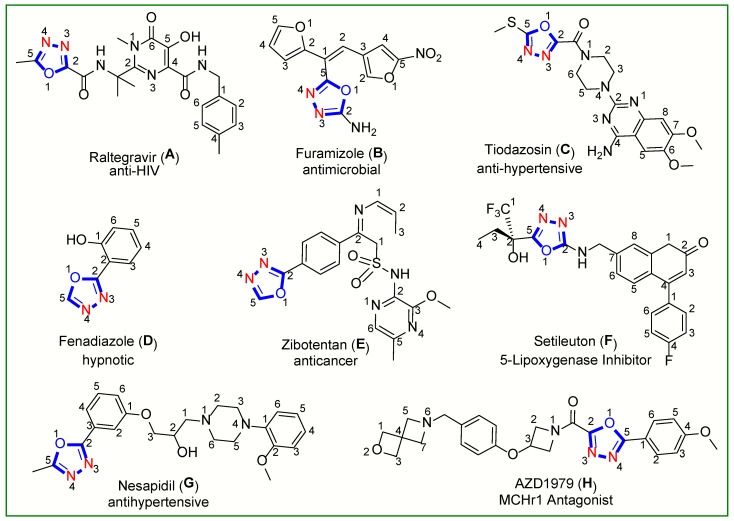
1,3,4-Oxadiazoles containing drugs.

Oxadiazoles are five-membered heterocyclic compounds that contain two nitrogen atoms and one oxygen atom. On the basis of the arrangement of nitrogen and oxygen atoms, there are four different isomers of oxadiazoles [1]. The isomeric 1,3,4-oxadiazole ring is generally advantageous in terms of thermal and metabolic stabilities, water solubility, as well as lipophilicity profiles [2,3]. 1,3,4-Oxadiazoles act as a flat aromatic linker in some cases, giving the molecule a better orientation to bind to biological targets [3]. Many drugs such as raltegravir (anti-HIV) (**A**), furamizole (antibacterial) (**B**), tidazosin (α_1_-adrenergic receptor antagonist) (**C**), fenadiazole (hypnotic) (**D**), zibotentan (anticancer) (**E**), setileuton (MK-0633; LOX inhibitor) (**F**), nesapidil (vasodialator) (**G**), and AZD1979 (melanin concentration receptor 1 antagonist) (**H**) contain a 1,3,4-oxodiazole ring in their structure (Figure 1) [4,5,6,7,8,9,10,11]. The anticancer [12,13,14], antitubercular [15,16], anti-HIV [17], antimicrobial [18], anti-inflammatory [19], anticonvulsant [20], and antimalarial [14] activities, as well as many other biological activities [1,21,22,23,24] reported in the literature, have drawn a lot of attention to 1,3,4-oxadiazole analogues.

Cancer, on the other hand, is one of the most pressing health concerns today, with nearly 10 million deaths and 19.3 million new cases reported worldwide in 2020. The number of new cancer cases is expected to exceed 28.4 million by 2040 [25]. Breast (2.26 million), lung (2.21 million), colon and rectum (1.93 million), and prostate (1.41 million) cancers were the most common cancer types reported in 2020 [26]. Chemotherapy is the most widely used and well-known cancer treatment. It is, however, linked to a number of negative consequences, hence the search for new compounds is always admired. Inspired by the miraculous biological activities of 1,3,4-oxadiazoles, we have synthesized eight new oxadiazole analogues and studied their antiproliferative effect against the nearly five dozen cancer cell lines, as well as their antibacterial potentials.

**Figure 2:**
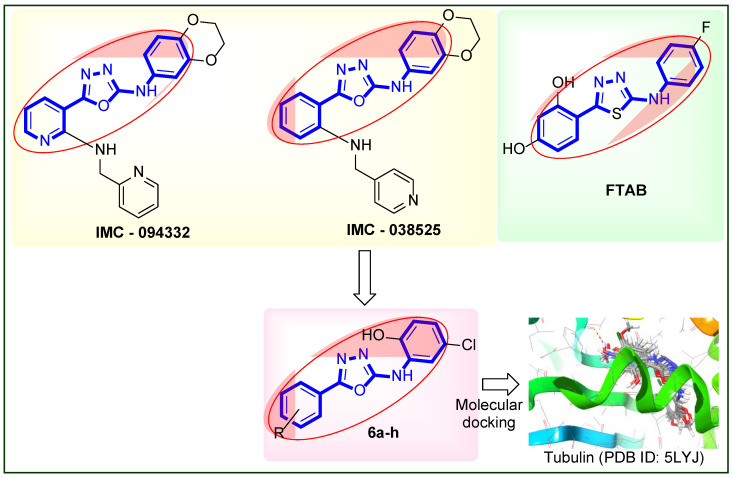
Design of new 2,5-disubstituted-1,3,4-oxadiazoles.

The oxadiazole-linked aryl core (represented as bold blue bond) of IMC-038525 (tubulin inhibitor; IC_50_ = 0.39 ± 0.06 μM), IMC-094332 (tubulin inhibitor), and FABT (anticancer; ID_50_ = 6.2 ± 1.4 (HCV29T), 3.9 ± 1.1 (SW707) and 4.2 ± 1.2 (T47D) µg/mL, respectively) was used in the design strategy for the newer oxadiazole analogues shown in Figure 2 [27,28,29]. Similarly, FTAB is another anticancer compound, whose 1,3,4-thiadiazole ring was replaced with an isostere 1,3,4-oxadiazole in the title compounds (**6a-h**). Microtubules are highly dynamic structures made up of α- and β-tubulin heterodimers that are indispensable for cell division (mitosis) and growth [30]. The expression of specific tubulin isotypes has been linked to cancer, but the exact mechanism is still unknown. Many tubulin-binding chemotherapeutics target the tubulin family protein to suppress the dynamics of the mitotic spindle to cause mitotic arrest and cell death [31]. Because the synthesized compounds have the same pharmacophoric core as the tubulin inhibitors IMC-038525 and IMC-094332, they can be used as tubulin inhibitors. As a result, a putative mechanism of action of the target compounds (**6a-h**) as anticancer agents was discovered using a molecular docking approach.

The oxadiazoles were also reported as antibacterial and antifungal agents [16,18,32,33]. Hence, we further explored their antibacterial activity as well as their molecular docking studies against DNA gyrase, a potential target for many antibacterial drugs. Since some of the compounds exhibited promising antibacterial activity, molecular docking against DNA gyrase was studied for the title compounds (**6a-h**).

## 2. Results

### 2.1. Chemistry

**Scheme 1:**
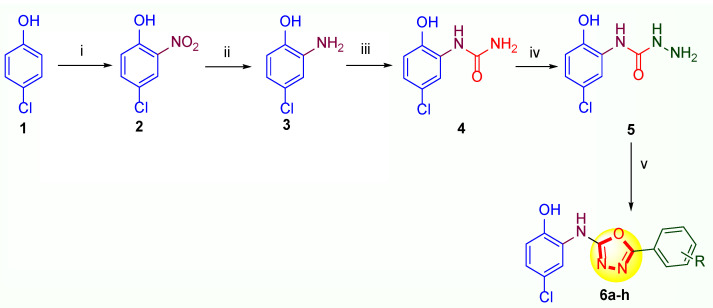
Reagents and conditions: (i) HNO_3_, H_2_SO_4_, 45 °C, 1 h, 82% yield; (ii) Sn, HCl, 100 °C, 1 h, 78%, yield; (iii) NaCNO, CH_3_COOH; 25 °C, 30 min, 88% yield; (iv) NH_2_NH_2_.H_2_O, C_2_H_5_OH, 80 °C, 24 h, 85% yield; (v) ArCHO, NaHSO_3_, C_2_H_5_OH-H_2_O (1:2), 100 °C, 8–10 h, 71–89% yield.

The synthesis of 2,5-disubstituted oxadiazoles was accomplished in five steps. The initial step involved the preparation of 4-chloro-2-nitrophenol (**2**) via the nitration of 4-chlorophenol (0.1 mol; ~12.86 g) with nitric acid (HNO_3_) and concentrated H_2_SO_4_. In the subsequent step, 4-chloro-2-nitrophenol (**2**) (75 mmol; 13.02 g) was reduced in the presence of Sn/HCl to obtain 2-amino-4-chlorophenol (**3**). 2-Amino-4-chlorophenol (**3**) (50 mmol; 7.18 g) was dissolved in 10 mL glacial acetic acid followed by the addition of 40 mL hot water. A solution of sodium cyanate (75 mmol; 4.88 g) in hot water was then mixed to a solution of 2-amino-4-chlorophenol (**3**) with continuous stirring with a magnetic stirrer for 30 min at room temperature to obtain 1-(5-chloro-2-hydroxyphenyl)urea (**4**), which was then refluxed with hydrazine hydrate (60 mmol; 3.00 mL) in absolute ethanol for 24 h to obtain N-(5-chloro-2-hydroxyphenyl)hydrazinecarboxamide (**5**) [34,35]. In the final step, an equimolar mixture of compound (**5**) (1 mmol; 201 mg) and aromatic aldehyde (1 mmol) was refluxed in water/ethanol solvent (2:1) and 10% mol of sodium bisulphite for 8–10 h to obtain the final product: 4-chloro-2-((5-aryl-1,3,4-oxadiazol-2-yl)amino)phenol analogues (**6a-h**) [36]. The reaction sequences are summarized in Scheme 1. The final compounds were characterized via spectroscopic techniques (nuclear magnetic resonance and mass spectral analysis). The ^1^H NMR spectra of the prototype compounds **6a** showed two doublets at δ ppm 6.85 and 7.01 for the two aromatic protons (H3 and H4), respectively, while a singlet at δ ppm 7.98 was observed for another proton (H5) of the chlorophenyl ring. Two multiplets at δ ppm 7.13–7.31 (H3’ and H4’) and 7.70–7.75 (H2’ and H6’) were observed for four protons of the 4-fluorophenyl ring. Two singlets at δ ppm 8.76 and 11.05 were observed for the secondary amine (ArNH) and phenolic (ArOH) functions, respectively. The ^13^C NMR of the compound exhibited twelve peaks at δ ppm 152.60 (oxadiazole C), 148.66 (oxadiazole C), 148.11 (C–F), 144.54 (C−OH), 141.39 (C1), 128.64 (C2’ and C6’), 125.66 (C−Cl), 122.81 (C4), 122.41 (C1’), 117.17 (C6), 115.51 (C3), and 108.54 (C3’ and C5’). The molecular mass (ESI-MS) peak [M + 1]^+^ was observed at 306.01, while the isotopic peak [M + 3]^+^ was observed at 308.00. The HPLC chromatogram of one of the compounds (**6h**) was recorded at λ_210_ nm and a retention time of 4.19 min demonstrated a purity level of >99% (Please refer to the Supplementary Material, Figure S28).

### 2.2. Anticancer Activity

All the compounds were designed based on the pharmacophore of tubulin inhibitors, and their anticancer activity was accessed using the National Cancer Institute protocol (NCI US) reported elsewhere [37,38,39,40]. The title compounds were initially screened via one dose assay at 10 µM drug concentration against 56 NCI cancer cell lines, and their results in the form of growth percent (GP) and percent growth inhibition (PGI) are given in Table 1 and Table 2, respectively. The GP and PGI are related as PGI = 100 − GP. Compounds **6b**, **6e**, and **6g** showed maximum sensitivity against PC-3 (PGI = 14.13), NCI-H460 (PGI = 19.55), and NCI-H522 (PGI = 11.4), respectively, while compound **6a** was found to be sensitive against NCI-H522 (PGI = 23.32). Compound **6d** displayed maximum sensitivity against breast cancer cell line, MCF7 (PGI = 24.79), and MDA-MB-468 (PGI = 26.01), while compound 6f demonstrated maximum sensitivity against UACC-62 (PGI = 21.25), NCI-H522 (PGI = 20.32), and HCT-116 (PGI = 20.15). Compound **6h** showed maximum anticancer activity among the series and was found to be sensitive against SNB-19 (PGI = 65.12), NCI-H460 (PGI = 55.61), and SNB-75 (PGI = 54.68). Compound **6h** demonstrated <50 to >40 PGI against eight cancer cell lines including NCI/ADR-RES (PGI = 48.28), NCI-H226 (PGI = 42.59), HOP-62 (PGI = 40.28), SF-295 (PGI = 45.26), SF-539 (PGI = 43.79), OVCAR-4 (PGI = 40.03), MDA-MB-231 (PGI = 42.58), and HS 578T (PGI = 47.22), while it demonstrated <40 to >20 PGI against nine cancer cell lines including HCT-116 (PGI = 35.59), H322M (PGI = 33.58), A459 (PGI = 34.85), SF-268 (PGI = 29.96), NCI- ACNH (PGI = 27.99), U251 (PGI = 26.04), SN 12C (PGI = 23.24), NCI-H23 (PGI = 20.82), and 786-O (PGI = 20.42) [Table 2]. The average PGI of individual panels was calculated and compared with the anticancer activity of the reference drug imatinib and their results are given in Table 3. Compound **6h** exhibited far better anticancer activity against non-small cancer, colon, CNS, melanoma, ovarian, renal, and breast cancer cell lines. The data for imatinib were retrieved from the NCI website with NSC Code 759854 [37]. The structure–activity relationship was established based on the anticancer activity. The 3,4,5-trimethoxy (**6h**) substitution of the phenyl ring exhibited promising anticancer activity followed by 2-hydroxy (**6f**) substitution. The overall structure–activity relationship (SAR) can be represented as 3,4,5-trimethoxy > 2-hydroxy > 4-nitro > 4-hydroxy-3-methoxy substitution in the phenyl ring directly attached to the oxadiazole ring.

### 2.3. Molecular Docking

**Figure 3:**
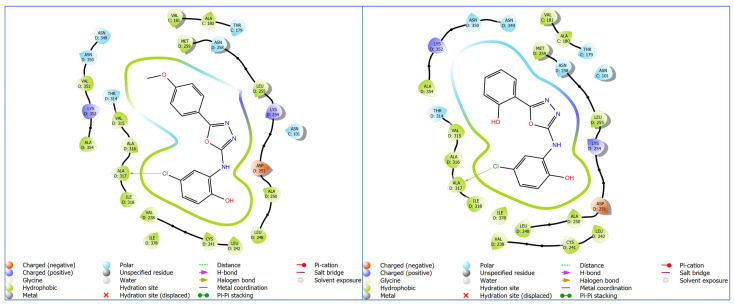
Two-dimensional binding interaction of ligands **6d** and **6f** against the active site of tubulin–combretastatin A4 (PDB ID: 5LYJ).

**Figure 4:**
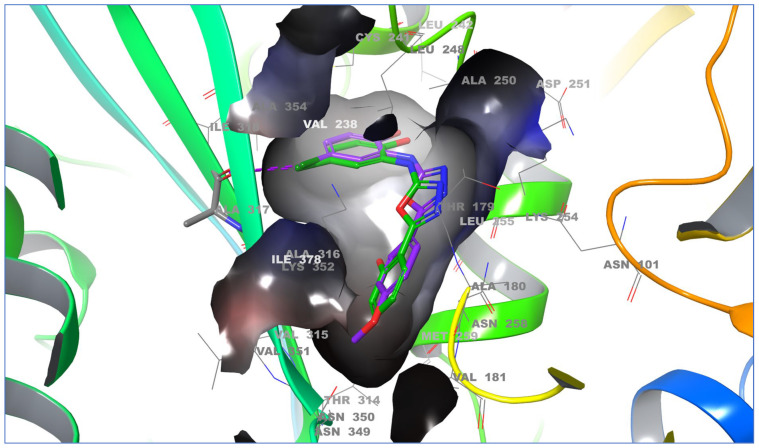
Three-dimensional binding interaction of ligands **6d** (violet) and **6f** (green)with the active site of tubulin–combretastatin A4 (PDB ID: 5LYJ).

**Figure 5:**
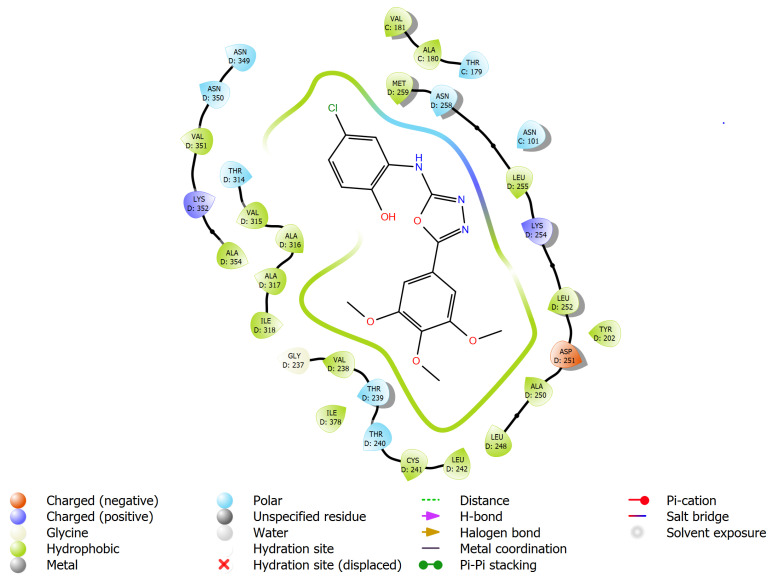
Two-dimensional binding interaction of ligand **6h** with the active site of tubulin–combretastatin A4 (PDB ID: 5LYJ).

Some of the oxadiazole analogues displayed good anticancer activity against some of the cancer cell lines and the compounds were also designed based on the pharmacophore of tubulin inhibitors, hence a molecular docking study of tubulin was explored against the active site of tubulin–combretastatin A4 (PDB ID: 5LYJ). The molecular docking scores and interactions are given in Table 4. The molecular docking score of the ligands (**6a-h**) ranged from −7.617 to −8.389 kcal/mol and they displayed two types of interactions, including H-bonding and halogen bonding. Ligand **6d** displayed maximum binding affinity with a docking score of −8.389 kcal/mol and exhibited a halogen bond interaction with the residue Ala317, while ligand **6e** displayed a docking score of −7.617 kcal/mol with no significant interaction. Ligands **6d** and **6f** displayed a similar type of interaction with the residue Ala317 through a halogen bond interaction. The 2D and 3D interactions of ligands **6d** and **6f** are shown in Figure 3 and Figure 4, respectively. Ligands **6a** and **6b** displayed a H-bond interaction with the residue Asn258, while they showed a halogen bond interaction with the residues Ala317 and Asn349, respectively. Ligand **6h** displayed no significant electrostatic interaction; however, the trimethoxy phenyl ring lies with the hydrophobic cavity of containing residues Leu252, Ala250, Leu248, Leu242, Cys241, Val238, Ile318, Ala317, and Ala316, as shown in Figure 5.

### 2.4. Antibacterial Activity

The antibacterial activity of compounds **6a-h** against Gram-negative (*E. coli*, NCIM 5051 and *P. aeruginosa*, ATCC 10145) as well as Gram-positive (*S. aureus*, NCIM 2079 and *B. subtilis*, NCIM 2439) bacterial strains was assessed via a disc diffusion method and broth dilution method as per the reported protocol [41,42,43]. Compounds **6d**, **6f**, **6g**, and **6h** displayed less antibacterial activity with zones of inhibition ranging from 4.8 ± 0.75 to 6.7 ± 0.20 mm at 200 µg/mL drug concentrations and exhibited MICs ranging from 128 to 512 µg/mL against various bacterial strains. Compounds **6a** and **6d** displayed moderate antibacterial activity with MICs ranging between 16 and 64 µg/mL and zones of inhibition ranging between 13.0 ± 0.21 and 9.8 ± 0.25 mm at 200 µg/mL. Compound 6e displayed good antibacterial activity against *S. aureus*, *E. coli*, and *P. aeruginosa* with MICs of 8 µg/mL, while it inhibited B. subtilis with an MIC of 8 µg/mL and a zone of inhibition ranging between 14.0 ± 0.45 and 16.0 ± 0.26 mm at 200 µg/mL. Compound **6c** displayed the most promising antibacterial activity against both the Gram-negative as well as Gram-positive bacteria with MICs of 8 µg/mL and zones of inhibition ranging from 17.0 ± 0.40 to 17.0 ± 0.15 mm at 200 µg/mL; however, the standard drug ciprofloxacin exhibited MICs of 8 µg/mL. The antibacterial activity in terms of zones of inhibition and MICs is given in Table 5. The 4-nitro substitution exhibited promising antibacterial activity followed by 2-chloro substitution in the phenyl ring attached to the oxadiazole ring. The SAR of the antibacterial activity can be represented as 4-nitro > 2-chloro > 4-fluoro > 4-chloro substitution in the phenyl ring. Molecular docking studies of the molecular target DNA gyrase were carried out to explore the binding mode of compounds against the 2-oxo-1,2-dihydroquinoline derivative binding active site of DNA gyrase (PDB ID; 6KZV). The results of the molecular docking studies are given in Table S1 and the 2D binding interactions of ligands **6c** and **6e** are given in Figure S1 (refer to Supplementary Materials). The antibacterial activity was supported by in silico studies, and the compounds (**6c** and **6e**) that had the maximum docking score (of −6.200 and −6.424 kcal/mol, respectively) demonstrated promising antibacterial activity.

## 3. Discussion

The 1,3,4-oxadiazoles were designed using the pharmacophore found in some tubulin inhibitors (IMC-094322 and IMC-038525) and the anticancer agent FTAB. The current research aimed to create scaffolds with the basic pharmacophore of tubulin inhibitors (IMC-094322 and IMC-038525) and a low-molecular-weight small compound. Molecular docking studies were performed for the tubulin–combretastatin A4 binding site of the D-chain (PDB ID: 5LYJ). We discovered that the benzene ring substitution produced higher docking scores than IMC-094322 and IMC-038525; hence, we performed modifications in the benzene ring rather than the dioxane ring of IMC-094322 and IMC-038525. The oxadiazole analogues were prepared in satisfactory yields and characterized using spectroscopic techniques followed by their anticancer evaluation at 10 µM against nine panel of 56 cancer cell lines as per the NCI US protocol. Some of the compounds displayed promising anticancer activity against a few cancer cell lines. Compound **6h** demonstrated significant anticancer activity against SNB-19 (CNS cancer), NCI-H460 (non-small-cell lung cancer), and SNB-75 (CNS cancer) with PGIs of 65.12, 55.61, and 54.68 percent, respectively at 10 µM. Compound **6h** also displayed efficient binding against the tubulin–combretastatin A4 binding site of the D-chain with a docking score of −8.030 kcal/mol and a trimethoxy phenyl ring lying within the hydrophobic cavity surrounded by important residues Leu252, Ala250, Leu248, Leu242, Cys241, Val238, Ile318, Ala317, and Ala316. The antibacterial activities of the oxadiazoles were also assessed against Gram-negative (*E. coli* and *P. aeruginosa*) as well as Gram-positive (*S. aureus* and *B. subtilis*) bacterial strains via a disc diffusion method and broth dilution method as per the reported protocol. Compounds **6c** and **6e** demonstrated good bacterial inhibition against both the Gram-positive as well as Gram-negative bacterial strains. A molecular docking study was conducted for the title compounds (**6a-h**) against DNA gyrase, one of the attractive molecular targets for many antibacterial drugs. All the compounds exhibited docking scores ranging between −5.883 and −6.424 kcal/mol. The binding affinities of compounds **6c** (docking score = −6.200 kcal/mol) and **6e** (docking score = −6.424 kcal/mol) were found to be the highest among the series, and these compounds also possessed promising antibacterial activity.

## 4. Materials and Methods

### 4.1. Chemistry

#### 4.1.1. Method for the Synthesis of 4-Chloro-2-nitrophenol (**2**)

An amount of 21 mL of concentrated H_2_SO_4_ and 15 mL of concentrated HNO_3_ was mixed in a 250 mL round-bottom flask with a few smaller pieces of porcelain. 4-Chlorophenol (100 mmol; 12.86 g) was added in portions of 2–3 g to the acidic mixture, shaking the reaction mixture after each addition. The temperature was controlled to no more than 55 °C by immersing the flask into the cold-water bath. The reaction mixture was then heated to 45 °C for 45 min with continuous shaking on magnetic stirrer [44]. The content of the flask was poured into the crushed ice with continuous stirring with a glass rod to obtain the precipitate. The precipitate was filtered, washed with water, and re-crystallized from methanol to obtain 4-chloro-2-nitrophenol (**2**). Colour, light yellow; yield, 82%; Mp 86–88 °C.

#### 4.1.2. Method for the Synthesis of 2-Amino-4-chlorophenol (**3**)

4-Chloro-2-nitrophenol (**2**) (75 mmol; 13.02 g) was dissolved in concentrated HCl (50 mL) and 45 g granulated tin was added, and the reaction mixture was left to heat up in a boiling water bath (100 °C) for 60 min [44]. The reaction mixture was poured into the crushed ice to obtain solid precipitate, which was filtered, dried, and re-crystallized with absolute ethanol to obtain 2-amino-4-chlorophenol (**3**). Colour, brown; yield, 78%; Mp 142–144 °C.

#### 4.1.3. Method for the Synthesis of 1-(5-Chloro-2-hydroxyphenyl)urea (**4**)

2-Amino-4-chlorophenol (**3**) (50 mmol; 7.18 g) was dissolved in 20 mL glacial acetic acid with heating and 40 mL hot water was added and 40 mL of NaCNO (75 mmol; 4.88 g) was added with continuous stirring at room temperature (25 °C) for 30 min. It was then allowed to stand for another 30 min and then dipped into cold-water bath to obtain precipitate, which was then filtered, dried, and re-crystallized with hot water to obtain light brown coloured precipitate of 1-(5-chloro-2-hydroxyphenyl)urea (**4**) [34,35]. Colour, light brown; yield, 88%; Mp 183–185 °C.

#### 4.1.4. Method for the Synthesis of N-(5-Chloro-2-hydroxyphenyl)hydrazinecarboxamide (**5**)

1-(5-chloro-2-hydroxyphenyl)urea (**4**) (40 mmol; 7.44 g) was refluxed at 80 °C with hydrazine hydrate (60 mmol; 3.00 mL) in absolute ethanol for 24 h to obtain *N*-(5-chloro-2-hydroxyphenyl)hydrazinecarboxamide (**5**) [34,35]. Colour, creamy; yield, 85%; Mp 202–204 °C.

#### 4.1.5. General Method for the Synthesis of 4-Chloro-2-((5-aryl-1,3,4-oxadiazol-2-yl)amino)phenol Analogues (**6a-h**)

An equimolar mixture of *N*-(5-chloro-2-hydroxyphenyl)hydrazinecarboxamide (**5**) (1 mmol, 201 mg) and aromatic aldehyde (1 mmol) was dissolved in 5 mL ethanol, then 5 mL water was added and the reaction mixture was allowed to reflux at 100 °C for 8–10 h with an addition of 10% mol NaHSO_3_ (5 mL). The reaction mixture was then concentrated and poured into the crushed ice, filtered, dried, and recrystallized with ethanol to obtain the final product, 4-chloro-2-((5-aryl-1,3,4-oxadiazol-2-yl)amino)phenol analogues (**6a-h**) [36].

*4-Chloro-2-((5-(4-fluorophenyl)-1,3,4-oxadiazol-2-yl)amino)phenol* (**6a**): yield: 78%; Mp 102–104 °C; ^1^H NMR (300 MHz, DMSO-*d*_6_): δ ppm: 6.85 (d, 1H, *J* = 6.0 Hz, ArH), 7.01 (d, 1H, *J* = 6.1 Hz, ArH), 7.13–7.31 (m, 2H, ArH), 7.70–7.75 (m, 2H, ArH), 7.89 (s, 1H, ArH), 8.76 (s, 1H, NH), 11.05 (s, 1H, ArOH); ^13^C NMR (75 MHz, DMSO-*d*_6_): δ ppm: 152.60, 148.66, 148.11, 144.54, 141.39, 128.64, 125.66, 122.81, 122.41, 117.17, 115.51, 108.54; Anal. Calc. for C_14_H_9_ClFN_3_O_2_: C, 55.01; H, 2.97; N, 13.75, found: C, 54.98; H, 2.99; N, 13.70%. ESI-MS m/z = 306.01 (M + 1)^+^, 308.00 (M + 3)^+^.

*4-Chloro-2-((5-(4-chlorophenyl)-1,3,4-oxadiazol-2-yl)amino)phenol* (**6b**): yield: 72%; Mp 154–156 °C; ^1^H NMR (300 MHz, DMSO-*d*_6_): δ ppm: 6.91 (d, 1H, *J* = 7.5 Hz, ArH), 7.03 (d, 1H, *J* = 9.0 Hz, ArH), 7.40 (s, 1H, ArH), 7.57 (d, 2H, *J* = 7.8 Hz, ArH), 8.11 (d, 2H, 3.6 Hz, ArH), 8.75 (s, 1H, NH), 11.00 (s, 1H, ArOH); ^13^C NMR (75 MHz, DMSO-*d*_6_): δ ppm: 152.59, 148.53, 143.51, 135.69, 134.52, 129.97, 128.99, 127.79, 124.49, 124.21, 118.19, 116.89; Anal. Calc. for C_14_H_9_Cl_2_N_3_O_2_: C, 52.20; H, 2.82; N, 13.04, found: C, 52.15; H, 2.80; N, 13.01%. ESI-MS m/z = 322.14 (M + 1)^+^, 324.12 (M + 3)^+^.

*4-Chloro-2-((5-(4-nitrophenyl)-1,3,4-oxadiaol-2-yl)amino)phenol* (**6c**): yield: 83%; Mp 196–198 °C; ^1^H NMR (300 MHz, DMSO-*d*_6_): δ ppm: 6.86 (d, 1H, *J* = 6.1 Hz, ArH), 6.91 (d, 1H, *J* = 6.1 Hz, ArH), 7.92 (d, 2H, *J* = 6.3 Hz, ArH), 8.08 (s, 1H, ArH), 8.27 (d, 2H, *J* = 6.9 Hz, ArH), 8.76 (s, 1H, NH), 11.37 (s, 1H, ArOH); ^13^C NMR (75 MHz, DMSO-*d*_6_): δ ppm: 152.12, 147.57, 145.01, 140.47, 139.02, 128.04, 127.37, 124.12, 122.62, 122.06, 118.04, 115.63; Anal. Calc. for C_14_H_9_ClFN_4_O_4_: C, 50.54; H, 2.73; N, 16.84, found: C, 50.49; H, 2.75; N, 16.81%. ESI-MS m/z = 333.23 (M + 1)^+^, 335.25 (M + 3)^+^.

*4-Chloro-2-((5-(4-methoxyphenyl)-1,3,4-oxadiazol-2-yl)amino)phenol* (**6d**): yield: 81%; Mp 162–164 °C; ^1^H NMR (300 MHz, DMSO-*d*_6_): δ ppm: 3.83 (s, 3H, OCH_3_), 6.72 (d, 1H, *J* = 6.1 Hz, ArH), 6.99 (d, 1H, *J* = 6.3 Hz, ArH), 7.13 (d, 2H, *J* = 6.0 Hz, ArH), 7.32 (s, 1H, ArH), 8.09 (d, 2H, *J* = 6.1 Hz, ArH), 9.81 (s, 1H, NH), 10.42 (s, 1H, ArOH); ^13^C NMR (75 MHz, DMSO-*d*_6_): 152.65, 148.52, 147.66, 142.57, 135.39, 127.24, 124.45, 118.13, 116.99, 115.97, 114.83, 56.18; Anal. Calc. for C_15_H_12_ClN_3_O_3_: C, 56.70; H, 3.81; N, 13.23, found: C, 56.67; H, 3.79; N, 13.21%. ESI-MS m/z = 318.11 (M + 1)^+^, 320.10 (M + 3)^+^.

*4-Chloro-2-((5-(2-chlorophenyl)-1,3,4-oxadiazol-2-yl)amino)phenol* (**6e**): yield: 71%; Mp 182–184 °C; ^1^H NMR (300 MHz, DMSO-*d*_6_): δ ppm: 6.84 (d, 1H, *J* = 6.0 Hz, ArH), 6.86 (d, 1H, *J* = 6.1 Hz, ArH), 7.41–7.45 (m, 2H, ArH), 7.51–7.54 (m, 2H, ArH), 7.91–7.94 (m, 1H, ArH), 8.10 (s, 1H, ArH), 8.78 (s, 1H, NH), 11.26 (s, 1H, ArOH); ^13^C NMR (75 MHz, DMSO-*d*_6_): 152.63, 148.59, 142.51, 136.92, 132.22, 130.13, 129.31, 128.92, 127.71, 127.34, 124.41, 118.11, 116.91; Anal. Calc. for C_14_H_9_Cl_2_N_3_O_2_: C, 52.20; H, 2.82; N, 13.04, found: C, 52.17; H, 2.80; N, 13.00%. ESI-MS m/z = 322.13 (M + 1)^+^, 324.19 (M + 3)^+^.

*4-Chloro-2-((5-(2-hydroxyphenyl)-1,3,4-oxadiazol-2-yl)amino)phenol* (**6f**): yield: 82%; Mp 168–170 °C; ^1^H NMR (300 MHz, DMSO-*d*_6_): δ ppm: 6.85 (d, 1H, *J* = 6.0 Hz, ArH), 6.93 (d, 1H, *J* = 6.1 Hz, ArH), 7.14–7.69 (m, 4H, ArH), 8.14 (s, 1H, ArH), 9.00 (s, 1H, NH), 10.25 (s, 1H, ArOH), 10.97 (s, 1H, ArOH); ^13^C NMR (75 MHz, DMSO- *d*_6_): 152.63, 148.95, 147.14, 142.51, 135.61, 130.11, 127.72, 126.30, 124.42, 121.81, 118.13, 117.82, 116.91, 108.11; Anal. Calc. for C_14_H_10_ClN_3_O_3_: C, 55.37; H, 3.32; N, 13.84, found: C, 55.34; H, 3.30; N, 13.81%. ESI-MS m/z = 304.23 (M + 1)^+^, 306.23 (M + 3)^+^.

*4-Chloro-2-((5-(4-hydroxy-3-methoxyphenyl)-1,3,4-oxadiazol-2-yl)amino)phenol* (**6g**): yield: 88%; Mp 112–114 °C; ^1^H NMR (300 MHz, DMSO-*d*_6_): δ ppm: 3.83 (s, 3H, OCH_3_), 6.82 (d, 1H, *J* = 6.0 Hz, ArH), 6.85 (d, 1H, *J* = 6.1 Hz, ArH), 7.31 (s, 2H, *J* = 6.0 Hz, ArH), 7.86 (s, 1H, ArH), 8.15 (s, 1H, ArH), 9.80 (s, 1H, NH), 10.48 (s, 1H, ArOH), 10.88 (s, 1H, ArOH); ^13^C NMR (75 MHz, DMSO-*d*_6_): 152.59, 148.65, 148.10, 144.53, 141.39, 128.64, 125.67, 122.80, 121.40, 121.23, 117.17, 115.51, 115.47, 108.55, 55.40; Anal. Calc. for C_15_H_12_ClN_3_O_4_: C, 53.99; H, 3.62; N, 12.59, found: C, 53.87; H, 3.60; N, 12.57%. ESI-MS m/z = 334.09 (M + 1)^+^, 336.06 (M + 3)^+^.

*4-Chloro-2-((5-(3,4,5-trimethoxyphenyl)-1,3,4-oxadiazol-2-yl)amino)phenol* (**6h**): yield: 89%; Mp 180–182 °C; ^1^H NMR (300 MHz, DMSO- *d*_6_): δ ppm: 3.69 (s, 3H, OCH_3_), 3.83 (s, 6H, OCH_3_), 6.87 (d, 1H, *J* = 6.1 Hz, ArH), 7.03 (d, 1H, *J* = 6.0 Hz, ArH), 7.47 (s, 1H, ArH), 7.88 (s, 2H, ArH), 8.94 (s, 1H, NH), 11.10 (s, 1H, ArOH); ^13^C NMR (75 MHz, DMSO- *d*_6_): 153.20, 152.42, 144.54, 138.79, 129.72, 128.48, 122.72, 121.46, 117.05, 115.43, 104.61, 103.62, 60.15, 55.82; Anal. Calc. for C_17_H_16_ClN_3_O_5_: C, 54.05; H, 4.27; N, 11.12, found: C, 54.02; H, 4.25; N, 11.10%. ESI-MS m/z = 378.27 (M + 1)^+^, 378.28 (M + 3)^+^. HPLC purity: 99.129%.

### 4.2. Anticancer Activity

The anticancer activity of the compounds was assessed using the standard NCI US protocol at 10 µM drug concentration [37,38,39,40]. In our earlier published work, we provided a thorough explanation of the experimental process. [45].

### 4.3. Molecular Docking Studies

The tubulin–combretastatin A4 complex’s X-ray crystallographic structure, with a resolution of 2.40 Å and an R-value of 0.192 (observed), was retrieved from protein data bank (PDB) [46]. The combretastatin A4 binding site of D-chain was used to prepare grid generation and molecular docking as per reported protocol [47].

The *E. coli* DNA gyrase complex with 2-oxo-1,2-dihydroquinoline derivative’s X-ray crystal structure with a resolution of 2.40 Å, R-value 0.199 (obs.), was retrieved from protein data bank (https://www.rcsb.org/structure/6KZV accessed on 16 March 2023) [48]. The standard molecular docking protocol was used for docking studies [49].

### 4.4. Antibacterial Activity

The antibacterial activities of the compounds were tested against *B. subtilis*, *E. coli*, *S. aureus*, and *P. aeruginosa* bacterial strains via disc diffusion method and broth dilution method [41,42,43] (please refer to the Supplementary Materials).

## 5. Conclusions

We discussed the design, synthesis, and anticancer activity of new 4-chloro-2-((5-aryl-1,3,4-oxadiazol-2-yl)amino)phenol analogues (**6a-h**) in the current investigation. The compounds were designed based on the pharmacophoric features of tubulin inhibitors (IMC-094322 and IMC-038525). Therefore, the compounds were further subjected to molecular docking studies against the active site of the tubulin–combretastatin A4 complex (PDB ID: 5LYJ); they displayed efficient binding with docking scores ranging between −7.617 and −8.389 kcal/mol and demonstrated two types of binding interactions: H-bond and halogen bond interactions. The compounds’ binding affinities against the active site of the tubulin–combretastatin A4 complex were found to be higher than those of the tubulin inhibitors IMC-094322 and IMC-038525 in the molecular docking studies, with the exception of compound **6e**. The compounds were prepared efficiently in five steps in good yields. The anticancer activity of the compounds was evaluated at 10 µM drug concentration as per the standard protocol. 4-Chloro-2-((5-(3,4,5-trimethoxyphenyl)-1,3,4-oxadiazol-2-yl)amino)phenol (**6h**) demonstrated significant anticancer activity against some of the cancer cell lines, including SNB-19, NCI-H460, and SNB-75 at 10 µM. Compound **6h** was discovered to be highly sensitive to the CNS cancer cell line SNB-19 (PGI = 65.12%), but less sensitive to the renal cancer cell line A498 (PGI = −30.65%). The average anticancer activity of compound **6h** against nine panels of cancer cell lines was compared with that of the reference drug imatinib at 10 µM. It was discovered that compound **6h** displayed significantly greater anticancer activity against non-small cancer, colon, CNS, melanoma, ovarian, renal, and breast cancer cell lines than imatinib. Based on anticancer activity, the SAR was concluded. It was discovered that the electron-donating group, such as 1,3,4-trimethoxy (compound **6h**; mean GP = 81.49%) and 2-hydroxy (compound **6f**; mean GP = 96.45%) substitution, as well as the electron-withdrawing 4-nitro (compound **6c**; mean GP = 96.97%) substitution on the phenyl ring demonstrated significant anticancer activity compared to other substitutions. The 4-chloro (compound **6b**; mean GP = 98.2%) substitution showed slightly more anticancer activity than 2-chloro (compound **6e**; mean GP = 100.79%) and 4-fluoro (compound **6a**; mean GP = 100.91%) substitution on the phenyl ring. Additionally, the 1,3,4-oxadiazoles’ antibacterial activities are well documented in the literature. The antibacterial activity of the compounds was studied against Gram-negative (*E. coli* and *P. aeruginosa*) as well as Gram-positive (*S. aureus* and *B. subtilis*) bacterial strains via the disc diffusion method and broth dilution method. The compounds 4-chloro-2-((5-(4-nitrophenyl)-1,3,4-oxadiazol-2-yl)amino)phenol (**6c**) and 4-chloro-2-((5-(2-chlorophenyl)-1,3,4-oxadiazol-2-yl)amino)phenol (**6e**) displayed the most promising antibacterial activities against both the Gram-negative as well as Gram-positive bacteria. Compound **6c** displayed the most significant antibacterial activities with MICs of 8 µg/mL and a zone of inhibition ranging from 17.0 ± 0.40 to 17.0 ± 0.15 mm at 200 µg/mL; however, the standard drug ciprofloxacin exhibited MICs of 4 µg/mL. The electron-withdrawing group, such as 4-nitro and 2-chloro substitutions on the phenyl ring, demonstrated the most promising antibacterial activity among the series; 4-chloro and 4-fluoro substitutions exhibited moderate antibacterial activity, while the electron-releasing group, such as 4-methoxy, 2-hydroxy, 4-hydroxy-3-methoxy, and 1,3,4-trimethoxy substitutions on the phenyl ring, displayed less antibacterial activity. A molecular docking study was performed for the title compounds (**6a-h**) against DNA gyrase (PDB ID: 6KZV), one of the most appealing targets for many antibacterial agents, and it was discovered that both compounds **6c** and **6e** had efficient binding affinity with docking scores of −6.200 and −6.424 kcal/mol, respectively. The results of the molecular docking studies revealed a correlation between docking scores and antibacterial activity. We have reported the anticancer and antibacterial activities of 1,3,4-oxadiazoles in the present investigation. The bioactivities of the compounds were found to be significant and some of the in vitro experimental results were found to be in good agreement with in silico studies. The current discovery could pave the way for future breakthroughs in cancer and bacterial disease treatment. The biological activities of these oxadiazoles can be increased through structural modification. In our laboratory, we are currently conducting additional research into the anticancer and antibacterial potential of modified oxadiazoles.

## Figures and Tables

**Table 1:** The anticancer activity of oxadiazoles at 10 µM in terms of growth percent (GP).

Panel	Cell Lines	6a	6b	6c	6d	6e	6f	6g	6h
*Leukemia*	CCRF-CEM	95.31	90.61	97.41	98.1	99.52	98.76	83.84	95.14
	HL-60(TB)	91.94	96.58	103.03	98.47	103.4	99.51	119.06	111.48
	K-562	93.01	91.61	96.74	94.05	97.96	92.24	100.44	95.61
	MOLT-4	93.23	94.59	100.15	95.64	96.06	96.19	91.33	103.1
	RPMI-8226	92.5	97.08	91.83	92.27	92.84	93.76	92.45	102.57
	SR	95.73	90.53	93.7	98.04	93.42	102.11	101.26	98.87
*Non-small cell lung cancer*	A549/ATCC	94.38	87.15	92.88	93.37	94.35	89.37	93.03	65.15
	EKVX	102.36	94	91.25	99.04	105.1	96.06	91.13	82
	HOP-62	105.99	102.74	97.05	101.95	105.83	104.42	94.4	59.72
	HOP-92	120.92	119.71	100.76	117.37	117.06	119.62	119.37	115.91
	NCI-H226	105.72	107.83	99.08	97.66	109.28	99.25	93.86	57.41
	NCI-H23	101.36	97.21	89.12	100.38	96.67	95.13	93.66	79.18
	NCI-H322M	99.79	98.15	97.07	101.97	101.53	98.01	102.39	66.42
	NCI-H460	104.48	101.02	104.92	105.25	80.45	99.18	102.16	44.39
	NCI-H522	76.68	86.11	87.33	85.94	82.27	79.68	88.6	82.71
*Colon cancer*	HCC-2998	99	104.62	105.4	112.79	106.12	95.16	107.17	86
	HCT-116	104.58	103.43	99.6	99.17	89.72	79.85	93.39	64.41
	HCT-15	95.67	95.25	93.24	95.62	91.28	87.97	95.78	93.96
	HT29	99.63	92.61	98.93	101.07	98.03	88.79	102.91	89.66
	KM12	99.8	99.76	99.97	100.21	98.14	99.38	100.05	99.39
	SW-620	103.18	95.56	101.61	106.56	96.99	98.15	97.46	87.4
*CNS cancer*	SF-268	101.69	103.23	98.7	99.96	102.59	95.4	96.32	70.04
	SF-295	94.74	94.54	93.94	95.86	97.97	90.94	96.1	54.74
	SF-539	96.75	102.67	94.36	97.09	97.61	99.35	96.03	56.21
	SNB-19	96.86	95.17	93.49	95.69	101.78	96.14	101.73	34.88
	SNB-75	106	104.3	88.1	110.01	102.53	85.25	95.7	45.32
	U251	108.42	101.95	106.8	101.81	110.38	98.86	101.29	73.96
*Melanoma*	LOX IMVI	105.56	96.63	92.71	105.81	95.88	99.59	93.23	93.26
	MALME-3M	99.56	94.69	95.31	98.67	99.03	96.85	91.74	87.03
	M14	101.31	101.56	104.02	108.72	96.97	102.72	101.42	102.06
	MDA-MB-435	98.9	92.79	103.33	103.96	95.59	97.75	108.74	89.29
	SK-MEL-28	103.13	107.19	101.02	105.24	109.05	101.02	118.11	99.89
	SK-MEL-5	98.18	99.34	97.51	97.81	100.18	96.24	100.75	94.83
	UACC-257	97.67	87.48	95.1	102.94	102.24	95.07	89.74	89.39
	UACC-62	86	86.98	87.06	86.4	89.93	78.75	83.7	95.39
*Ovarian cancer*	IGROV1	106.78	110.01	98.18	103.66	112.29	99.07	111.38	95.15
	OVCAR-3	108.43	106.72	105.56	108.82	109.51	107.84	113.11	93.72
	OVCAR-4	106.23	92.66	105.72	112.65	107.87	98.46	97.77	59.97
	OVCAR-5	96.14	100.93	95.05	96.83	103.38	95.35	97.86	99.22
	NCI/ADR-RES	100.27	94.74	100.36	103.69	99.8	98.12	97.93	51.72
	SK-OV-3	117.64	102.62	99.89	112.57	115.3	105.56	104.71	81.81
*Renal cancer*	786–0	94.95	98.08	100.57	100.28	108.08	92.93	111.45	79.58
	A498	113.51	103.52	117.23	121.53	127.23	104.1	130.65	101.82
	ACHN	106.84	107.22	103.61	105.68	101.8	106.06	103.15	72.01
	CAKI-1	103.12	95.64	87.11	94.99	98.39	88.89	92.01	81.17
	RXF 393	112.18	107.36	108.62	112.07	117.5	94.92	106.85	90.15
	SN 12C	92.59	96.79	91.49	101.31	101.82	97.73	95.3	76.76
	UO-31	92.04	93.53	85.65	92.2	104.92	84.53	81.99	82.82
*Prostate cancer*	PC-3	94.36	85.87	89.66	90.97	92.45	88.87	82.21	89.9
	DU-145	104.69	102.62	106.63	106.77	101.93	104.47	109.31	87.7
*Breast cancer*	MCF7	88.75	83.92	65.54	82.19	75.21	85.96	84.23	81.18
	MDA-MB-231	107.86	97.25	95.31	93.6	104.79	99.02	90.49	57.42
	HS 578T	111.42	100.53	100.25	105.9	109.39	103.85	93.65	52.78
	BT-549	115.53	112.05	108.47	111.75	101.27	104.77	111.86	98
	T-47D	106.36	102.08	95.87	111.22	102.18	97.96	83.03	81.98
	MDA-MB-468	101.42	90.64	73.99	87.2	91.04	100.03	108.19	82.26
Mean Growth Percent		100.91	98.2	96.97	101.09	100.79	96.45	98.85	81.49

**Table 2:** The comparative anticancer activity of oxadiazoles on percent growth inhibition (PGI) scale.

Compounds	Cell Line (Percent Growth Inhibition; % GI)			
	69.99 to 50.00	49.99 to 40.00	39.99 to 20.00	19.99 to 10.00
**6a**	−	−	NCI-H522 (23.32)	CCRF-CEM (16.16) UACC-62 (14) MCF7 (11.25)
**6b**	−	−	−	MCF7 (16.08) PC-3 (14.13) NCI-H522 (13.89) UACC-62 (13.02) A549 (12.85) UACC-257 (12.52)
**6c**	−	−	MCF7 (34.46)	UO-31 (14.35) UACC-62 (12.94) CAKI-1 (12.89) NCI-H522 (12.67) NCI-H23 (10.88) PC-3 (10.34)
**6d**	−	−	MDA-MB-468 (26.01) MCF7 (24.79)	MCF7 (17.81) NCI-H522 (14.06) UACC-62 (13.06) MDA-MB-468 (12.08)
**6e**	−	−	−	NCI-H460 (19.55) NCI-H522 (17.73) HCT-116 (10.28) UACC-62 (10.07)
**6f**	−	−	UACC-62 (21.25) NCI-H522 (20.32) HCT-116 (20.15)	UO-31 (15.47) SNB-75 (14.75) MCF7 (14.04) HCT-15 (12.03) HT29 (11.21) PC-3 (11.13) CAKI-1 (11.11) A549 (10.63)
**6g**	−	−	−	UO-31 (18.01) PC-3 (17.79) T-47D (16.97) UACC-62 (16.3) MCF7 (15.77) NCI-H522 (11.4) UACC-257 (10.26)
**6h**	SNB-19 (65.12) NCI-H460 (55.61) SNB-75 (54.68)	NCI/ADR-RES (48.28) HS 578T (47.22) SF-295 (45.26) SF-539 (43.79) NCI-H226 (42.59) MDA-MB-231 (42.58) HOP-62 (40.28) OVCAR-4 (40.03)	A459 (34.85) HCT-116 (35.59) NCI-H322M (33.58) SF-268 (29.96) ACNH (27.99) U251 (26.04) SN 12C (23.24) NCI-H23 (20.82) 786-O (20.42)	CAKI-1 (18.83) MCF7 (18.82) SK-OV-3 (18.19) T-47D (18.02) MDA-MB-468 (17.74) NCI-H522 (17.29) UO-31 (17.18) HCC-2998 (14.0) MALME-3M (12.97) DU-145 (12.3) MDA-MB-435 (10.71) UACC-257 (10.61) HT29 (10.34) PC-3 (10.1)

(−) None of the cell lines demonstrated this level of growth inhibition at 10 µM.

**Table 3:** The comparative anticancer activity of oxadiazoles and imatinib at 10 µM in terms of average PGI.

Panels	6a	6b	6c	6d	6e	6f	6g	6h	Imatinib
*Leukemia*	6.38	6.5	2.86	3.91	2.8	2.91	1.94	−1.13	9
*Non-Small Cancer Cell*	−1.29	0.68	4.50	−0.33	0.83	2.14	2.38	27.46	15.68
*Colon Cancer*	−0.31	1.46	0.21	−2.57	3.29	8.45	0.54	13.19	5.34
*CNS Cancer*	−1.29	0.68	4.50	−0.33	0.83	2.14	2.38	27.46	5.8
*Melanoma*	1.21	4.17	2.99	−1.19	1.39	4.00	1.57	6.11	−0.87
*Ovarian Cancer*	−5.92	−1.28	−0.79	−6.37	−8.03	−0.73	−3.79	19.74	−7.16
*Renal Cancer*	−2.18	−0.31	0.82	−4.01	−8.53	4.41	−3.06	16.53	3.25
*Prostate Cancer*	0.48	5.76	1.86	1.13	2.81	3.33	4.24	11.2	12.5
*Breast Cancer*	−5.22	2.26	10.09	1.36	2.69	1.40	4.76	24.39	12.15

**Table 4:** The molecular docking studies of oxadiazoles against tubulin.

S. No.	Compound	PDB ID: 5LYJ		
		Docking Score	Emodel Score	Interaction
1	**6a**	−8.233	−68.717	H-bond (Asn258, 2.52 Å); halogen bond (Ala317, 3.17 Å)
2	**6b**	−8.247	−71.236	H-bond (Asn258, 2.52 Å); halogen bond (Asn349, 3.43 Å)
3	**6c**	−8.356	−74.181	H-bond (Asn258, 2.58 Å)
4	**6d**	−8.389	−73.546	Halogen bond (Ala317, 3.35 Å)
5	**6e**	−7.617	−63.671	−
6	**6f**	−8.073	−71.277	Halogen bond (Ala317, 3.13 Å)
7	**6g**	−8.266	−74.933	H-bond (Asn349, 2.39 Å); halogen bond (Ala317, 3.12 Å)
8	**6h**	−8.030	−71.237	−
9	IMC-094322	−7.669	−42.387	−
10	IMC-038525	−7.941	−52.924	π-Cationic (Lys352, 6.46 Å)

(−) In molecular docking, no electrostatic interaction was seen.

**Table 5:** The antibacterial activity of oxadiazoles.

S. No.	Compound	Zone of Inhibition in mm at 200 µg/mL				Minimum Inhibitory Concentration (µg/mL)			
		*S. aureus*	*B. subtilis*	*E. coli*	*P. aeruginosa*	*S. aureus*	*B. subtilis*	*E. coli*	*P. aeruginosa*
1	**6a**	12.0 ± 0.36	11.0 ± 0.25	13.0 ± 0.21	12.0 ± 0.26	16	32	16	16
2	**6b**	11.0 ± 0.65	9.9 ± 0.30	11.0 ± 0.36	9.8 ± 0.25	32	64	32	64
3	**6c**	17.0 ± 0.40	17.0 ± 0.21	17.0 ± 0.15	17.0 ± 0.21	8	8	8	8
4	**6d**	5.5 ± 0.50	4.8 ± 0.75	5.6 ± 0.32	5.1 ± 0.15	256	512	256	256
5	**6e**	16.0 ± 0.49	14.0 ± 0.45	16.0 ± 0.26	16.0 ± 0.40	8	32	8	8
6	**6f**	5.2 ± 0.25	5.0 ± 0.10	5.4 ± 0.21	5.8 ± 0.10	256	256	256	256
7	**6g**	5.6 ± 0.35	5.0 ± 0.23	5.8 ± 0.30	5.6 ± 0.21	256	256	256	256
8	**6h**	6.7 ± 0.20	5.7 ± 0.29	6.0 ± 0.21	6.0 ± 0.23	128	256	128	128
9	Ciprofloxacin	18.0 ± 0.65	17.0 ± 0.26	18.0 ± 0.32	18.0 ± 0.31	4	4	4	4

## Data Availability

The Supplementary Material contains the information that supports the findings of this study.

## Supplementary Materials

The following supporting information can be downloaded at https://www.mdpi.com/article/10.3390/molecules28166086/s1, Figures S1 and S2: 2D and 3D binding interactions of ligands **6c** and **6e** against DNA gyrase; Figures S3–S10: Anticancer data of compounds **6a-h** against 56 cancer cell lines; Figures S11–S27: ^1^H NMR, ^13^C NMR and mass spectra of some of the synthetic compounds (**6a-h**); Figure S28: HPLC chromatogram of compound **6h**; Table S1: The molecular docking studies of oxadiazoles against DNA gyrase (PDB ID: 6KZV).

## Sample Availability

Samples of the compounds are available from the authors.
