# Supplementary material for: Synthesis and Anticancer Evaluation of 4-Chloro-2-((5-aryl-1,3,4-oxadiazol-2-yl)amino)phenol Analogues: An Insight into Experimental and Theoretical Studies
Source: Molecules. 2023 Aug 16;28(16):6086. doi: 10.3390/molecules28166086 (PMC10459877; doi:10.3390/molecules28166086)
Supplement: Supplementary file 1 [file molecules-28-06086-s001.zip › molecules-2509635-supplementary.pdf]

Synthesis and Anticancer Evaluation of 4-Chloro-2-((5-Aryl-1,3,4-Oxadiazol-2-yl)Amino)Phenol Analogues: an Insight into Experimental and Theoretical Studies

Obaid Afzal [1,*], Amena Ali [2], Abuzer Ali [3], Abdulmalik Saleh Alfawaz Altamimi [1], Manal A. Alossaimi [1], Md Afroz Bakht [4], Salahuddin [5], Mubarak A. Alamri [1], Md. Faiyaz Ahsan [6,*] and Mohamed Jawed Ahsan [7,*]

[1] Department of Pharmaceutical Chemistry, College of Pharmacy, Prince Sattam Bin Abdulaziz University, Al-Kharj 11942, Saudi Arabia
[2] Department of Pharmaceutical Chemistry, College of Pharmacy, Taif University, P.O. Box 11099, Taif 21944, Saudi Arabia
[3] Department of Pharmacognosy, College of Pharmacy, Taif University, P.O. Box 11099, Taif 21944, Saudi Arabia
[4] Department of Chemistry, College of Science and Humanity Studies, Prince Sattam Bin Abdulaziz University, Al-Kharj 11942, Saudi Arabia
[5] Department of Pharmaceutical Chemistry, Noida Institute of Engineering and Technology, (Pharmacy Institute), Knowledge Park-2, Greater Noida 201 306, India
[6] Department of Chemistry, Bihar National College, Patna 800 004, India
[7] Department of Pharmaceutical Chemistry, Maharishi Arvind College of Pharmacy, Jaipur 302 039, India
* Correspondence: obaid263@gmail.com (O.A.); faiyaz.ahsan123@gmail.com (M.F.A.); jawedpharma@gmail.com (M.J.A.)

SUPPLEMENTARY MATERIALS

Table S1. The molecular docking studies of oxadiazoles against DNA gyrase (PDB ID: 6KZV).

S. No.	Compound	PDB ID: 6KZV		
		Docking score	Emodel score	Interaction
1	**6a**	−6.014	−57.310	–
2	**6b**	−5.883	−58.996	H-bond (Asp73, and Gly77)
3	**6c**	−6.200	−61.495	Salt bridge (Arg76 and Arg136)
4	**6d**	−5.982	−57.022	H-bond (Asp73, and Gly77)
5	**6e**	−6.424	−57.902	H-bond (Glu50)
6	**6f**	−6.023	−57.897	H-bond (Asp73, and Gly77)
7	**6g**	−5.984	−58.883	H-bond (As73, and Gly77)
8	**6h**	−6.110	−56.457	H-bond (Asp73, Arg136, and Gly77); π-Cationic (Arg76)

Materials and Methods

Antibacterial activity

The disc diffusion method and broth dilution method were used to test antibacterial activity of the test compounds (**6a-j**) [41-43]. A stock solution of 1 mg/mL was prepared by dissolving 10 mg of the test compound in 10 ml of DMSO (1% v/v) and a 200 μg/mL dilution of the test compound was prepared by mixing 2 ml of the stock solution with 8 ml of DMSO (1% v/v). By combining 5.12 ml of the stock solution with 4.88 ml of DMSO (1% v/v), a 512 μg/mL dilution of the test compound was prepared. The two-fold dilution technique was used to prepare dilutions of 256, 128, 64, 32, 8, 4, and 2 μg/mL in similar way. Stock solutions of the compounds were made in accordance with the recommended volume range by dissolving

10 mg of the test compound in 100 μL DMSO and increasing the volume to 10 ml with artificial seawater. This was done to prevent toxicity from potential DMSO toxicity [43].

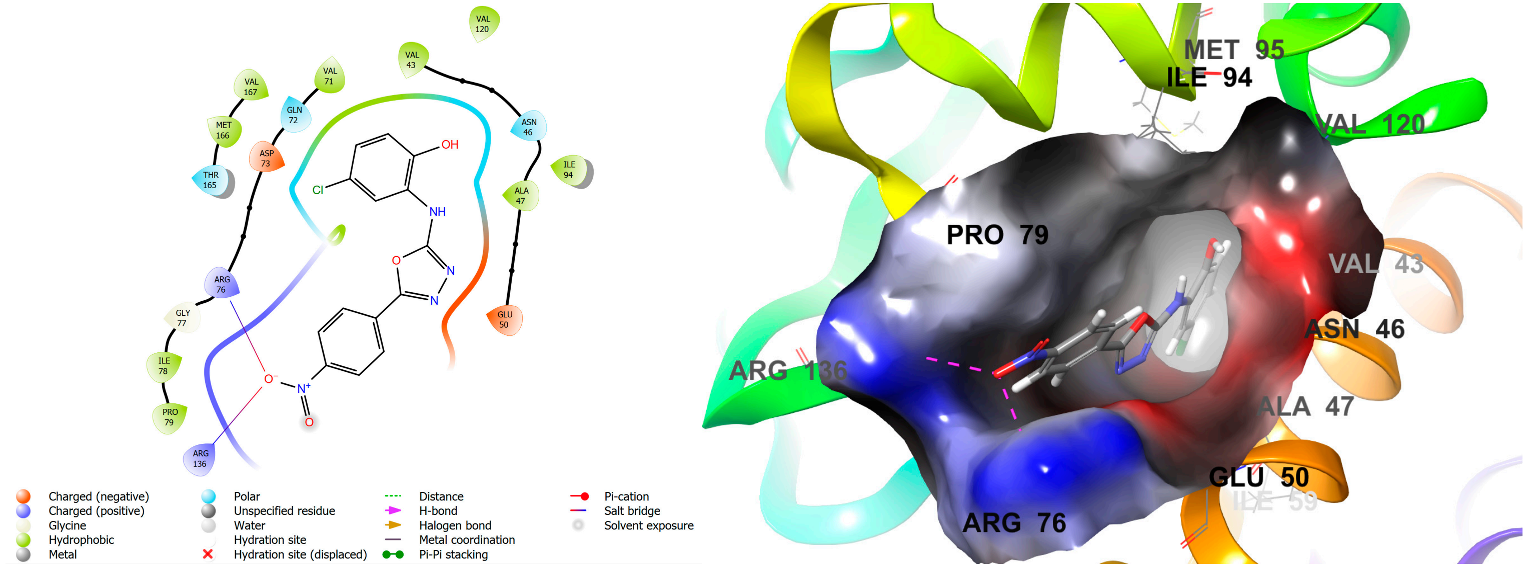

Figure S1. 2D and 3D binding interaction of ligands **6c** against DNA gyrase (PDB ID: 6KZV).

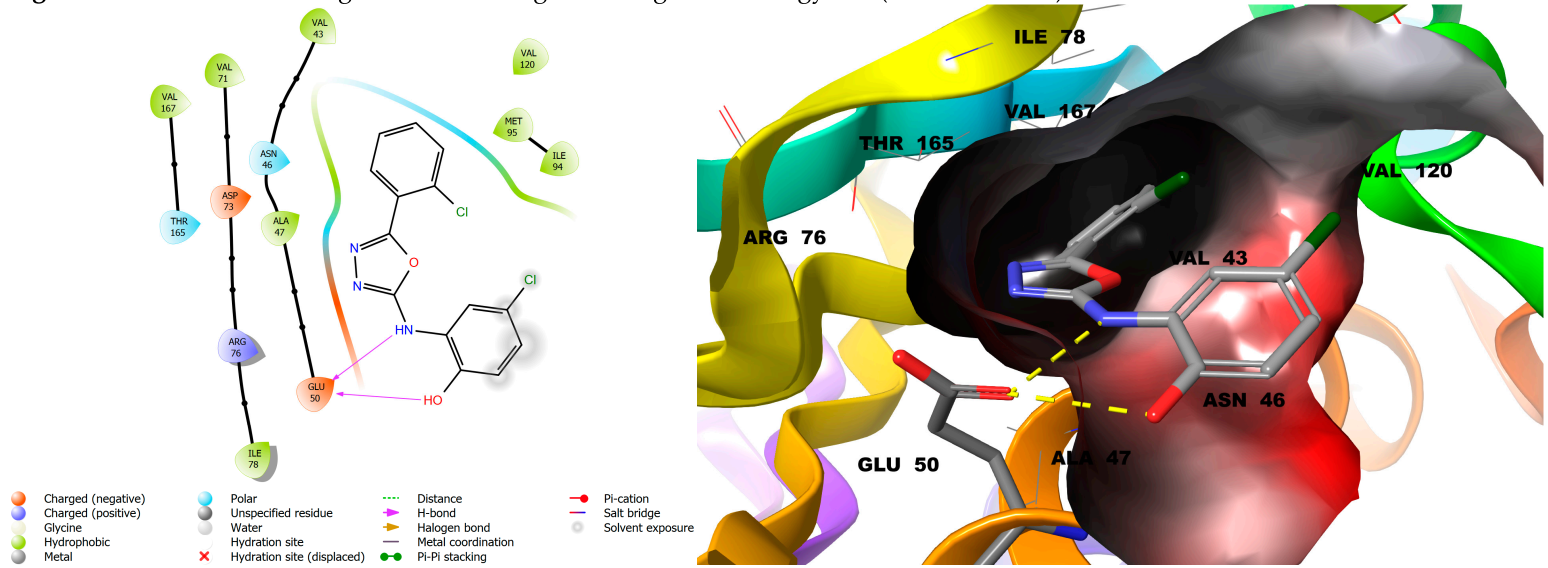

Figure S2. 3D binding interaction of ligands **6e** against DNA gyrase (PDB ID: 6KZV).

Developmental Therapeutics Program One Dose Mean Graph	NSC: D-839785 / 1	Conc: 1.00E-5 Molar	Test Date: Oct 24, 2022
	Experiment ID: 2210OS64		Report Date: Dec 22, 2022

Panel/Cell Line	Growth Percent
Leukemia	
CCRF-CEM	95.31
HL-60(TB)	91.94
K-562	93.01
MOLT-4	93.23
RPMI-8226	92.50
SR	95.73
Non-Small Cell Lung Cancer	
A549/ATCC	94.38
EKVX	102.36
HOP-62	105.99
HOP-92	120.92
NCI-H226	105.72
NCI-H23	101.36
NCI-H322M	99.79
NCI-H460	104.48
NCI-H522	76.68
Colon Cancer	
HCC-2998	99.00
HCT-116	104.58
HCT-15	95.67
HT29	99.63
KM12	99.80
SW-620	103.18
CNS Cancer	
SF-268	101.69
SF-295	94.74
SF-539	96.75
SNB-19	96.86
SNB-75	106.00
U251	108.42
Melanoma	
LOX IMVI	105.56
MALME-3M	99.56
M14	101.31
MDA-MB-435	98.90
SK-MEL-28	103.13
SK-MEL-5	98.18
UACC-257	97.67
UACC-62	86.00
Ovarian Cancer	
IGROV1	106.78
OVCAR-3	108.43
OVCAR-4	106.23
OVCAR-5	96.14
NCI/ADR-RES	100.27
SK-OV-3	117.64
Renal Cancer	
786-0	94.95
A498	113.51
ACHN	106.84
CAKI-1	103.12
RXF 393	112.18
SN12C	92.59
UO-31	92.04
Prostate Cancer	
PC-3	94.36
DU-145	104.69
Breast Cancer	
MCF7	88.75
MDA-MB-231/ATCC	107.86
HS 578T	111.42
BT-549	115.53
T-47D	106.36
MDA-MB-468	101.42
Mean	100.91
Delta	24.23
Range	44.24

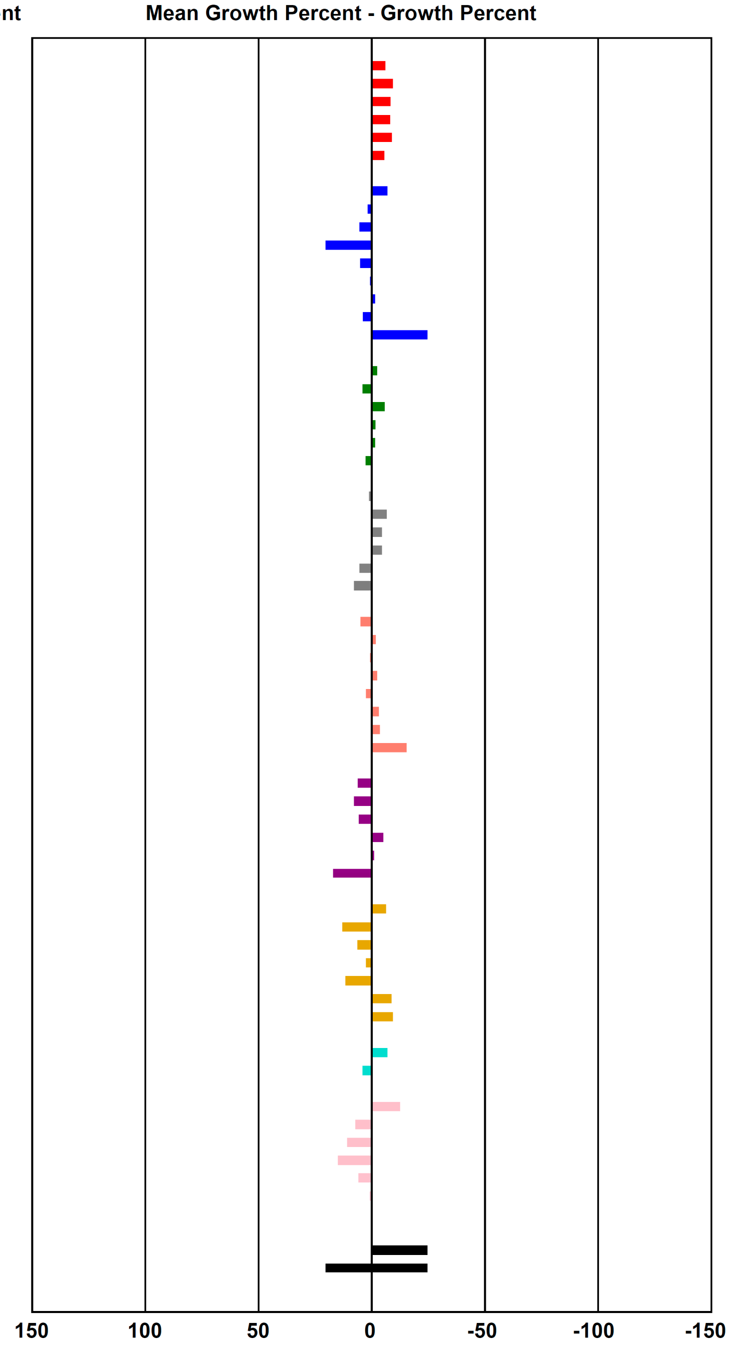

Figure S3. Anticancer data of compound **6a** against 56 cancer cell lines.

**Developmental Therapeutics Program**	**NSC:** D-839784 / 1	**Conc:** 1.00E-5 Molar	**Test Date:** Oct 24, 2022
**One Dose Mean Graph**	**Experiment ID:** 2210OS64		**Report Date:** Dec 22, 2022

Panel/Cell Line	Growth Percent
Leukemia	
CCRF-CEM	90.61
HL-60(TB)	96.58
K-562	91.61
MOLT-4	94.59
RPMI-8226	97.08
SR	90.53
Non-Small Cell Lung Cancer	
A549/ATCC	87.15
EKVX	94.00
HOP-62	102.74
HOP-92	119.71
NCI-H226	107.83
NCI-H23	97.21
NCI-H322M	98.15
NCI-H460	101.02
NCI-H522	86.11
Colon Cancer	
HCC-2998	104.62
HCT-116	103.43
HCT-15	95.25
HT29	92.61
KM12	99.76
SW-620	95.56
CNS Cancer	
SF-268	103.23
SF-295	94.54
SF-539	102.67
SNB-19	95.17
SNB-75	104.30
U251	101.95
Melanoma	
LOX IMVI	96.63
MALME-3M	94.69
M14	101.56
MDA-MB-435	92.79
SK-MEL-28	107.19
SK-MEL-5	99.34
UACC-257	87.48
UACC-62	86.98
Ovarian Cancer	
IGROV1	110.01
OVCAR-3	106.72
OVCAR-4	92.66
OVCAR-5	100.93
NCI/ADR-RES	94.74
SK-OV-3	102.62
Renal Cancer	
786-0	98.08
A498	103.52
ACHN	107.22
CAKI-1	95.64
RXF 393	107.36
SN12C	96.79
UO-31	93.53
Prostate Cancer	
PC-3	85.87
DU-145	102.62
Breast Cancer	
MCF7	83.92
MDA-MB-231/ATCC	97.25
HS 578T	100.53
BT-549	112.05
T-47D	102.08
MDA-MB-468	90.64
Mean	98.20
Delta	14.28
Range	35.79

Mean Growth Percent - Growth Percent

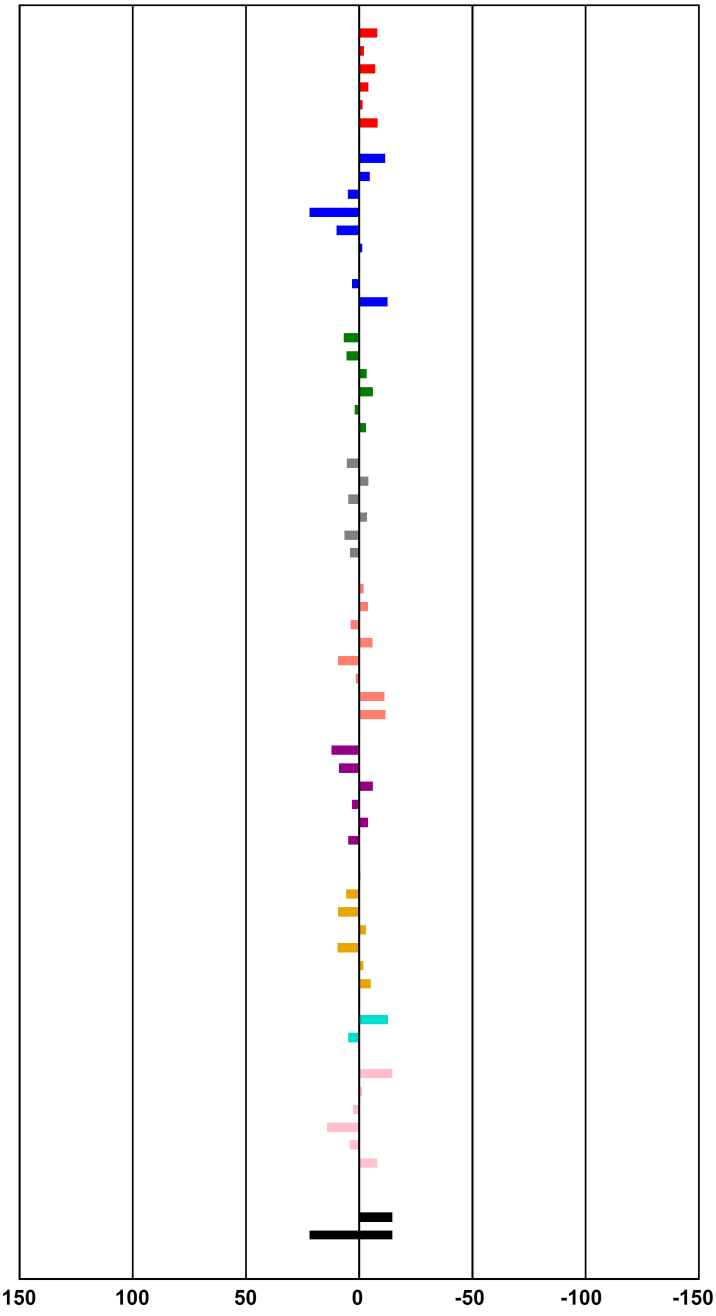

Figure S4. Anticancer data of compound **6b** against 56 cancer cell lines.

Developmental Therapeutics Program

One Dose Mean Graph

**NSC:** D-839786 / 1	**Conc:** 1.00E-5 Molar
**Test Date:** Oct 24, 2022	**Experiment ID:** 2210OS64
**Report Date:** Dec 22, 2022	

Panel/Cell Line	Growth Percent
Leukemia	
CCRF-CEM	97.41
HL-60(TB)	103.03
K-562	96.74
MOLT-4	100.15
RPMI-8226	91.83
SR	93.70
Non-Small Cell Lung Cancer	
A549/ATCC	92.88
EKVX	91.25
HOP-62	97.05
HOP-92	100.76
NCI-H226	99.08
NCI-H23	89.12
NCI-H322M	97.07
NCI-H460	104.92
NCI-H522	87.33
Colon Cancer	
HCC-2998	105.40
HCT-116	99.60
HCT-15	93.24
HT29	98.93
KM12	99.97
SW-620	101.61
CNS Cancer	
SF-268	98.70
SF-295	93.94
SF-539	94.36
SNB-19	93.49
SNB-75	88.10
U251	106.87
Melanoma	
LOX IMVI	92.71
MALME-3M	95.31
M14	104.02
MDA-MB-435	103.33
SK-MEL-28	101.02
SK-MEL-5	97.51
UACC-257	95.10
UACC-62	87.06
Ovarian Cancer	
IGROV1	98.18
OVCAR-3	105.56
OVCAR-4	105.72
OVCAR-5	95.05
NCI/ADR-RES	100.36
SK-OV-3	99.89
Renal Cancer	
786-0	100.57
A498	117.23
ACHN	103.61
CAKI-1	87.11
RXF 393	108.62
SN12C	91.49
UO-31	85.65
Prostate Cancer	
PC-3	89.66
DU-145	106.63
Breast Cancer	
MCF7	65.54
MDA-MB-231/ATCC	95.31
HS 578T	103.25
BT-549	108.47
T-47D	95.87
MDA-MB-468	73.99
Mean	96.97
Delta	31.43
Range	51.69

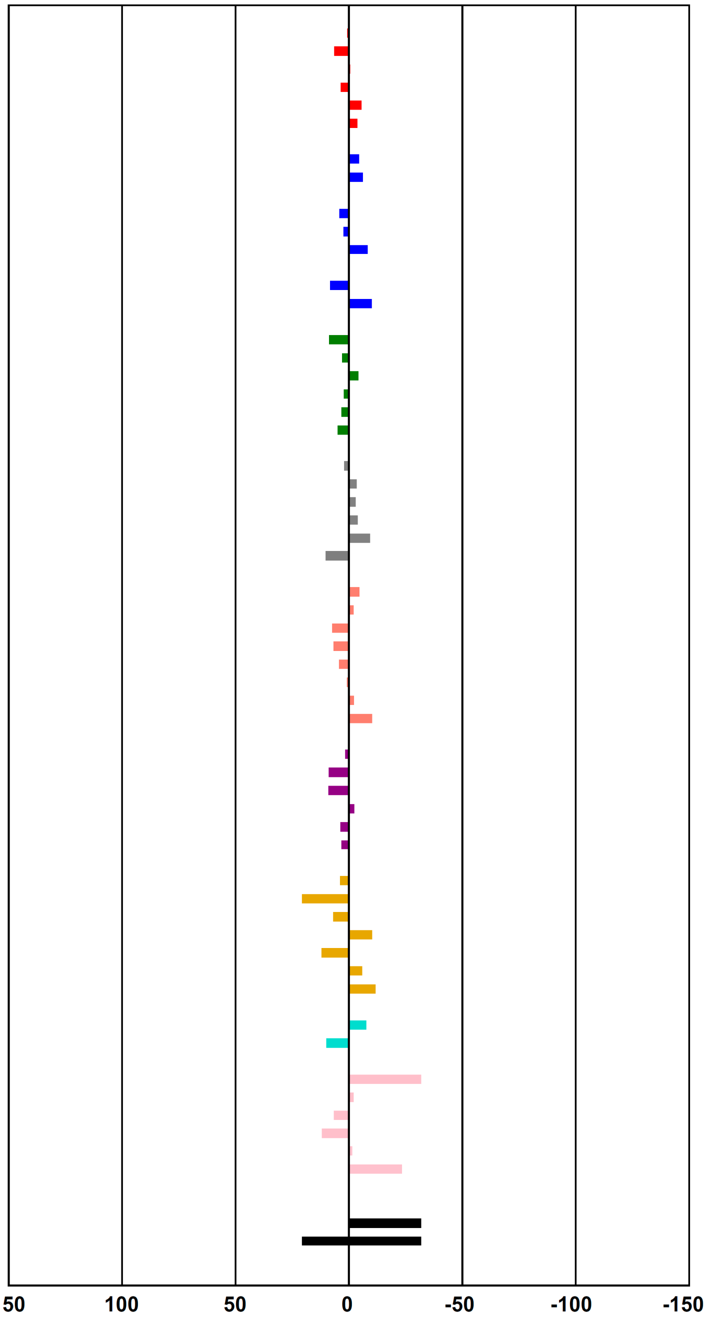

Figure S5. Anticancer data of compound **6c** against 56 cancer cell lines.

Developmental Therapeutics Program — One Dose Mean Graph

**NSC:** D-839787 / 1	**Conc:** 1.00E-5 Molar	**Test Date:** Oct 24, 2022	
**Experiment ID:** 2210OS64		**Report Date:** Dec 22, 2022	

Panel/Cell Line	Growth Percent
**Leukemia**	
CCRF-CEM	98.10
HL-60(TB)	98.47
K-562	94.05
MOLT-4	95.64
RPMI-8226	92.27
SR	98.04
**Non-Small Cell Lung Cancer**	
A549/ATCC	93.37
EKVX	99.04
HOP-62	101.95
HOP-92	117.37
NCI-H226	97.66
NCI-H23	100.38
NCI-H322M	101.97
NCI-H460	105.25
NCI-H522	85.94
**Colon Cancer**	
HCC-2998	112.79
HCT-116	99.17
HCT-15	95.62
HT29	101.07
KM12	100.21
SW-620	106.56
**CNS Cancer**	
SF-268	99.96
SF-295	95.86
SF-539	97.09
SNB-19	95.69
SNB-75	110.01
U251	101.81
**Melanoma**	
LOX IMVI	105.81
MALME-3M	98.67
M14	108.72
MDA-MB-435	103.96
SK-MEL-28	105.24
SK-MEL-5	97.81
UACC-257	102.94
UACC-62	86.40
**Ovarian Cancer**	
IGROV1	103.66
OVCAR-3	108.82
OVCAR-4	112.65
OVCAR-5	96.83
NCI/ADR-RES	103.69
SK-OV-3	112.57
**Renal Cancer**	
786-0	100.28
A498	121.53
ACHN	105.68
CAKI-1	94.99
RXF 393	112.07
SN12C	101.31
UO-31	92.20
**Prostate Cancer**	
PC-3	90.97
DU-145	106.77
**Breast Cancer**	
MCF7	82.19
MDA-MB-231/ATCC	93.60
HS 578T	105.90
BT-549	111.75
T-47D	111.22
MDA-MB-468	87.20
Mean	101.09
Delta	18.90
Range	39.34

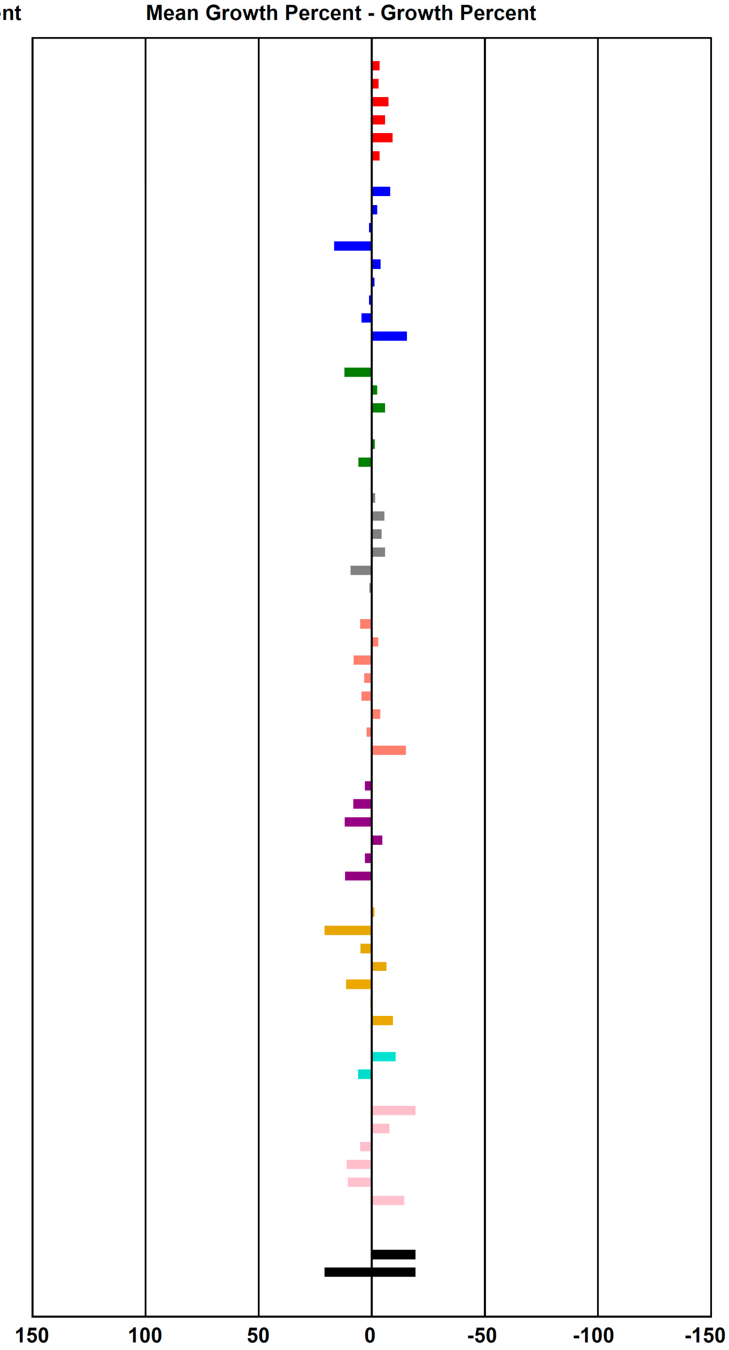

Figure S6. Anticancer data of compound **6d** against 56 cancer cell lines.

Developmental Therapeutics Program<br>One Dose Mean Graph	NSC: D-839788 / 1	Conc: 1.00E-5 Molar	Test Date: Oct 24, 2022
	Experiment ID: 2210OS64		Report Date: Dec 22, 2022

Panel/Cell Line	Growth Percent
Leukemia	
CCRF-CEM	99.52
HL-60(TB)	103.40
K-562	97.96
MOLT-4	96.06
RPMI-8226	92.84
SR	93.42
Non-Small Cell Lung Cancer	
A549/ATCC	94.35
EKVX	105.10
HOP-62	105.83
HOP-92	117.06
NCI-H226	109.28
NCI-H23	96.97
NCI-H322M	101.53
NCI-H460	80.45
NCI-H522	82.27
Colon Cancer	
HCC-2998	106.12
HCT-116	89.72
HCT-15	91.28
HT29	98.03
KM12	98.14
SW-620	96.99
CNS Cancer	
SF-268	102.59
SF-295	97.97
SF-539	97.61
SNB-19	101.78
SNB-75	102.53
U251	110.38
Melanoma	
LOX IMVI	95.88
MALME-3M	99.03
M14	96.97
MDA-MB-435	95.59
SK-MEL-28	109.05
SK-MEL-5	100.18
UACC-257	102.24
UACC-62	89.93
Ovarian Cancer	
IGROV1	112.29
OVCAR-3	109.51
OVCAR-4	107.87
OVCAR-5	103.38
NCI/ADR-RES	99.80
SK-OV-3	115.30
Renal Cancer	
786-0	108.08
A498	127.23
ACHN	101.80
CAKI-1	98.39
RXF 393	117.50
SN12C	101.82
UO-31	104.92
Prostate Cancer	
PC-3	92.45
DU-145	101.93
Breast Cancer	
MCF7	75.21
MDA-MB-231/ATCC	104.79
HS 578T	109.39
BT-549	101.27
T-47D	102.18
MDA-MB-468	91.04
Mean	100.79
Delta	25.58
Range	52.02

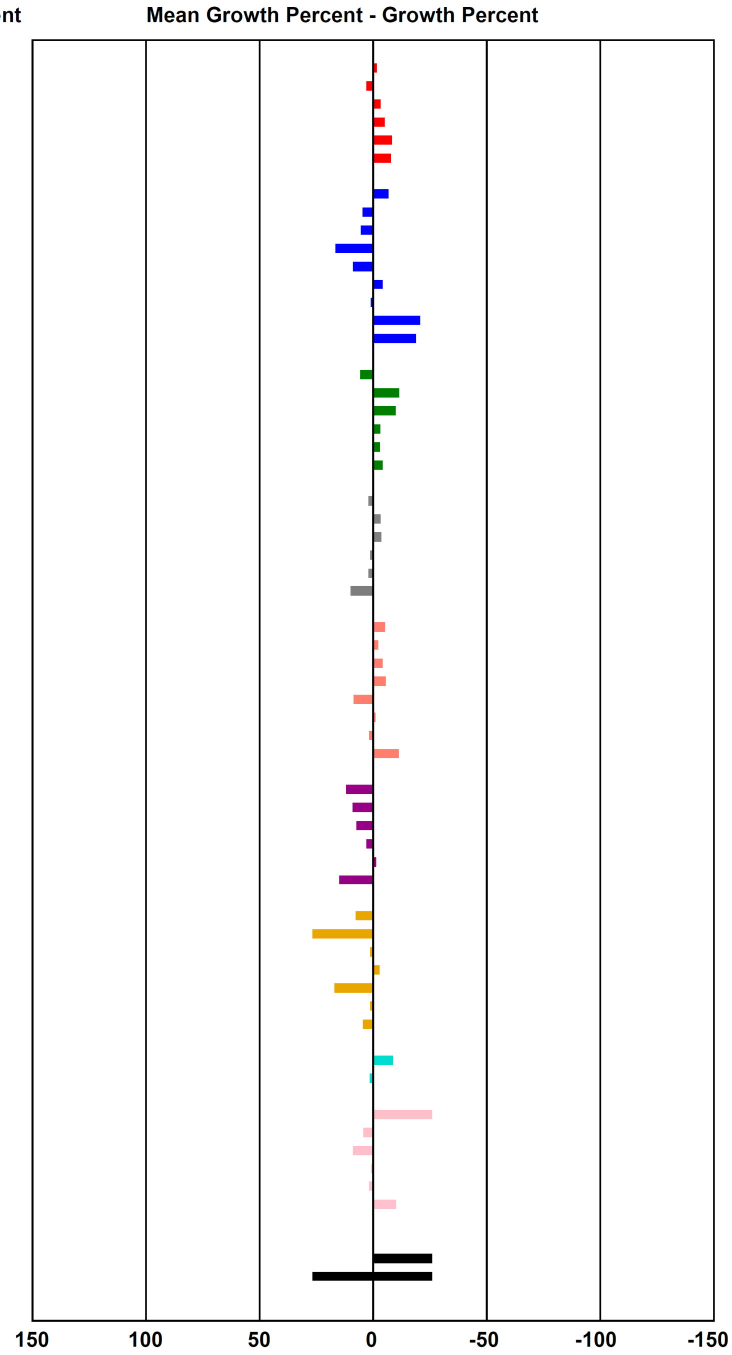

Figure S7. Anticancer data of compound **6e** against 56 cancer cell lines.

Developmental Therapeutics Program<br>One Dose Mean Graph	NSC: D-839790 / 1	Conc: 1.00E-5 Molar	Test Date: Oct 24, 2022
	Experiment ID: 2210OS64		Report Date: Dec 22, 2022

Panel/Cell Line	Growth Percent
Leukemia	
CCRF-CEM	98.76
HL-60(TB)	99.51
K-562	92.24
MOLT-4	96.19
RPMI-8226	93.76
SR	102.11
Non-Small Cell Lung Cancer	
A549/ATCC	89.37
EKVX	96.06
HOP-62	104.42
HOP-92	119.62
NCI-H226	99.25
NCI-H23	95.13
NCI-H322M	98.01
NCI-H460	99.18
NCI-H522	79.68
Colon Cancer	
HCC-2998	95.16
HCT-116	79.89
HCT-15	87.97
HT29	88.79
KM12	99.38
SW-620	98.15
CNS Cancer	
SF-268	95.40
SF-295	90.94
SF-539	99.35
SNB-19	96.14
SNB-75	85.25
U251	98.86
Melanoma	
LOX IMVI	99.59
MALME-3M	96.85
M14	102.72
MDA-MB-435	97.75
SK-MEL-28	101.02
SK-MEL-5	96.24
UACC-257	95.07
UACC-62	78.75
Ovarian Cancer	
IGROV1	99.07
OVCAR-3	107.84
OVCAR-4	98.46
OVCAR-5	95.35
NCI/ADR-RES	98.12
SK-OV-3	105.56
Renal Cancer	
786-0	92.93
A498	104.10
ACHN	102.06
CAKI-1	88.89
RXF 393	94.92
SN12C	97.73
UO-31	84.53
Prostate Cancer	
PC-3	88.87
DU-145	104.47
Breast Cancer	
MCF7	85.96
MDA-MB-231/ATCC	99.02
HS 578T	103.85
BT-549	104.77
T-47D	97.96
MDA-MB-468	100.03
Mean	96.45
Delta	17.70
Range	40.87

Mean Growth Percent - Growth Percent

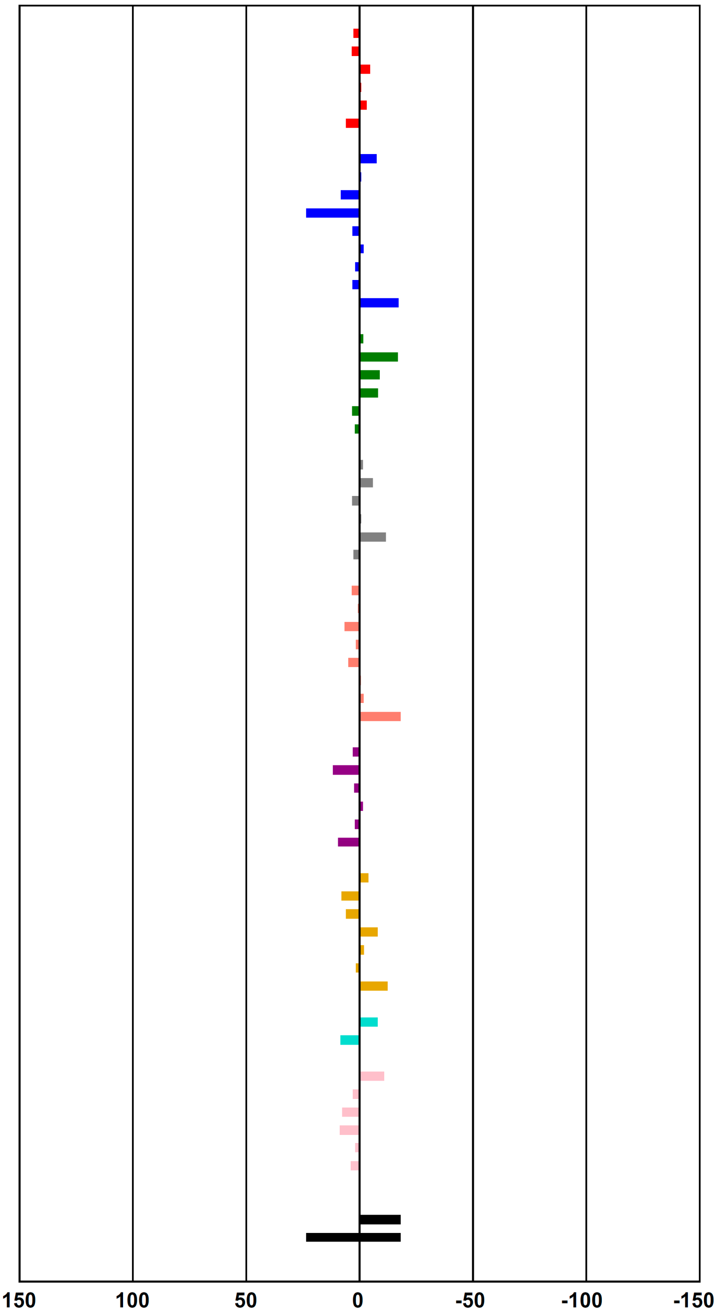

Figure S8. Anticancer data of compound **6f** against 56 cancer cell lines.

Developmental Therapeutics Program<br>One Dose Mean Graph	**NSC:** D-839783 / 1	**Conc:** 1.00E-5 Molar	**Test Date:** Oct 24, 2022
	**Experiment ID:** 2210OS64		**Report Date:** Dec 22, 2022

Panel/Cell Line	Growth Percent
**Leukemia**	
CCRF-CEM	83.84
HL-60(TB)	119.06
K-562	100.44
MOLT-4	91.33
RPMI-8226	92.45
SR	101.26
**Non-Small Cell Lung Cancer**	
A549/ATCC	93.03
EKVX	91.13
HOP-62	94.40
HOP-92	119.37
NCI-H226	93.86
NCI-H23	93.66
NCI-H322M	102.39
NCI-H460	102.16
NCI-H522	88.60
**Colon Cancer**	
HCC-2998	107.17
HCT-116	93.39
HCT-15	95.78
HT29	102.91
KM12	100.05
SW-620	97.46
**CNS Cancer**	
SF-268	96.32
SF-295	96.10
SF-539	96.03
SNB-19	101.73
SNB-75	95.70
U251	101.29
**Melanoma**	
LOX IMVI	93.23
MALME-3M	91.74
M14	101.42
MDA-MB-435	108.74
SK-MEL-28	108.11
SK-MEL-5	100.75
UACC-257	89.74
UACC-62	83.70
**Ovarian Cancer**	
IGROV1	111.38
OVCAR-3	113.11
OVCAR-4	97.77
OVCAR-5	97.86
NCI/ADR-RES	97.93
SK-OV-3	104.71
**Renal Cancer**	
786-0	111.45
A498	130.65
ACHN	103.15
CAKI-1	92.01
RXF 393	106.85
SN12C	95.30
UO-31	81.99
**Prostate Cancer**	
PC-3	82.21
DU-145	109.31
**Breast Cancer**	
MCF7	84.23
MDA-MB-231/ATCC	90.49
HS 578T	93.65
BT-549	111.86
T-47D	83.03
MDA-MB-468	108.19
Mean	98.85
Delta	16.86
Range	48.66

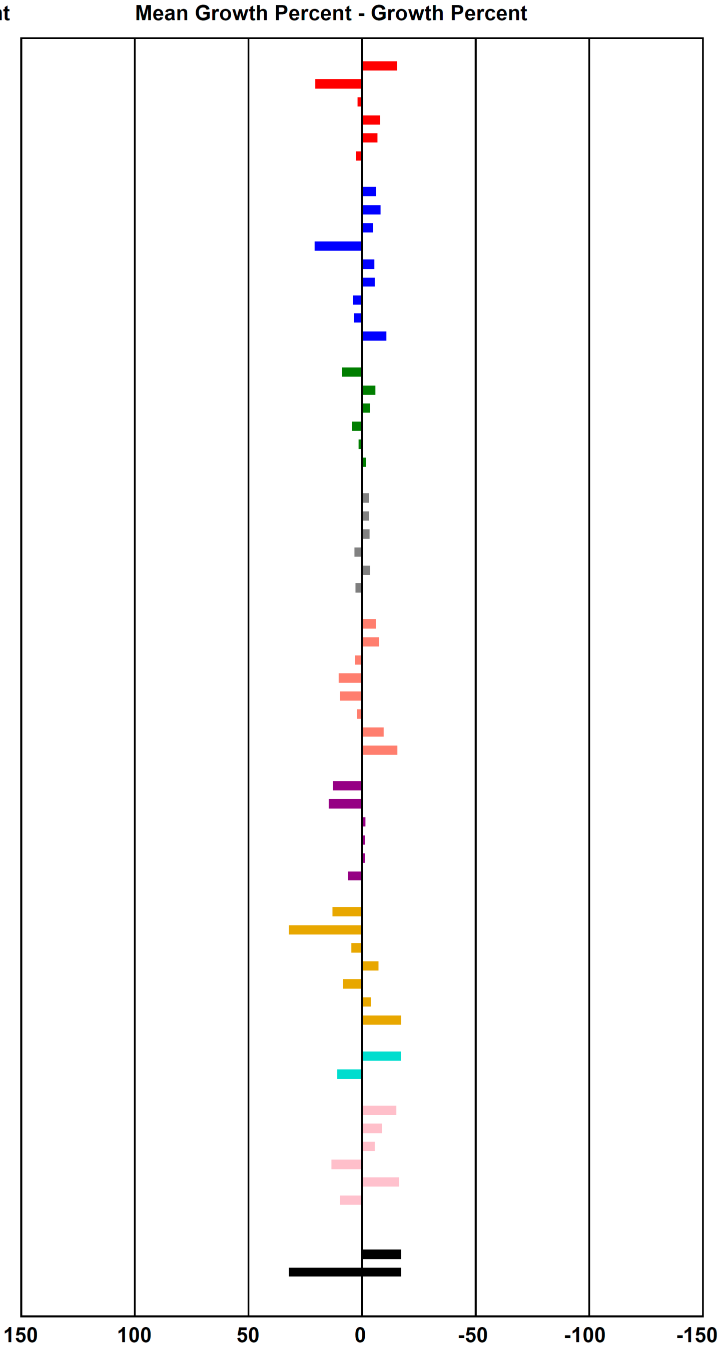

Figure S9. Anticancer data of compound **6g** against 56 cancer cell lines.

Developmental Therapeutics Program<br>One Dose Mean Graph	NSC: D-839782 / 1	Conc: 1.00E-5 Molar	Test Date: Oct 24, 2022
	Experiment ID: 2210OS64		Report Date: Jan 14, 2023

Panel/Cell Line	Growth Percent
**Leukemia**	
CCRF-CEM	95.14
HL-60(TB)	111.48
K-562	95.61
MOLT-4	103.10
RPMI-8226	102.57
SR	98.87
**Non-Small Cell Lung Cancer**	
A549/ATCC	65.15
EKVX	82.00
HOP-62	59.72
HOP-92	115.91
NCI-H226	57.41
NCI-H23	79.18
NCI-H322M	66.42
NCI-H460	44.39
NCI-H522	82.71
**Colon Cancer**	
HCC-2998	86.00
HCT-116	64.21
HCT-15	93.96
HT29	89.66
KM12	99.36
SW-620	87.40
**CNS Cancer**	
SF-268	70.04
SF-295	54.74
SF-539	56.21
SNB-19	34.88
SNB-75	45.32
U251	73.96
**Melanoma**	
LOX IMVI	93.26
MALME-3M	87.03
M14	102.06
MDA-MB-435	89.29
SK-MEL-28	99.89
SK-MEL-5	94.83
UACC-257	89.39
UACC-62	95.39
**Ovarian Cancer**	
IGROV1	95.15
OVCAR-3	93.72
OVCAR-4	59.97
OVCAR-5	99.22
NCI/ADR-RES	51.72
SK-OV-3	81.81
**Renal Cancer**	
786-0	79.58
A498	101.82
ACHN	72.01
CAKI-1	81.17
RXF 393	90.15
SN12C	76.76
UO-31	82.82
**Prostate Cancer**	
PC-3	89.90
DU-145	87.70
**Breast Cancer**	
MCF7	81.18
MDA-MB-231/ATCC	57.42
HS 578T	52.78
BT-549	98.00
T-47D	81.98
MDA-MB-468	82.26
Mean	81.49
Delta	46.61
Range	81.03

Mean Growth Percent - Growth Percent

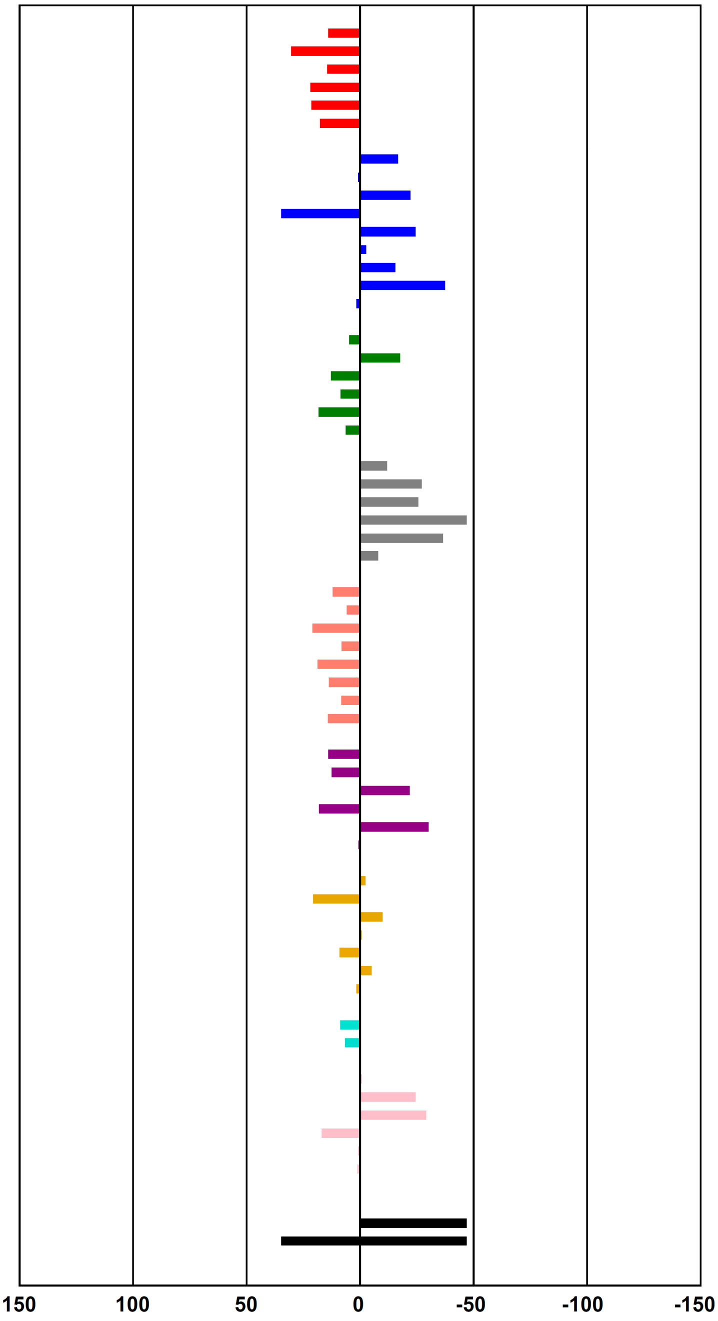

Figure S10. Anticancer data of compound **6h** against 56 cancer cell lines.

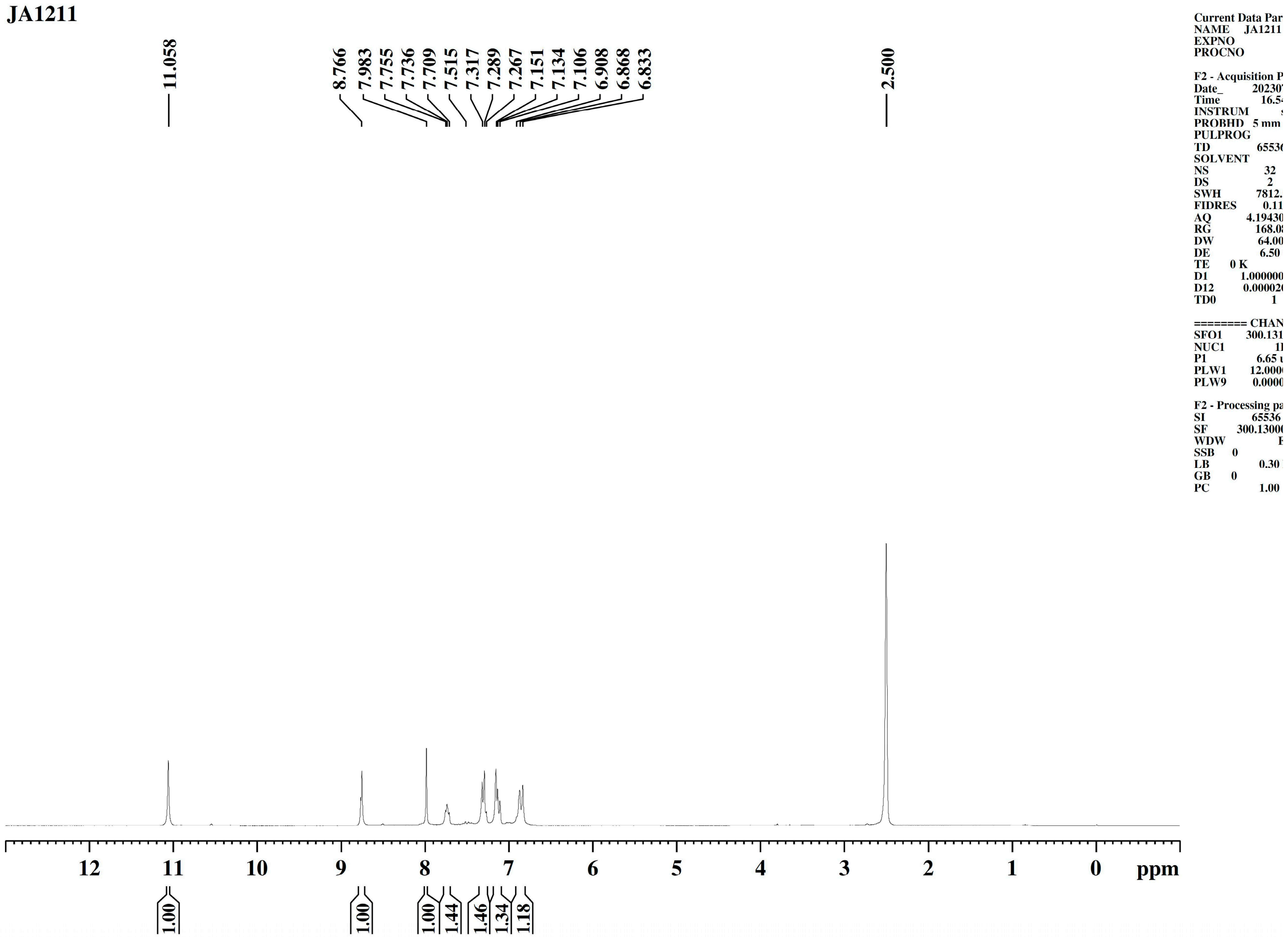

Figure S11. ^{1}H NMR spectra of 4-chloro-2-((5-(4-fluorophenyl)-1,3,4-oxadiazol-2-yl)amino)phenol (**6a**)

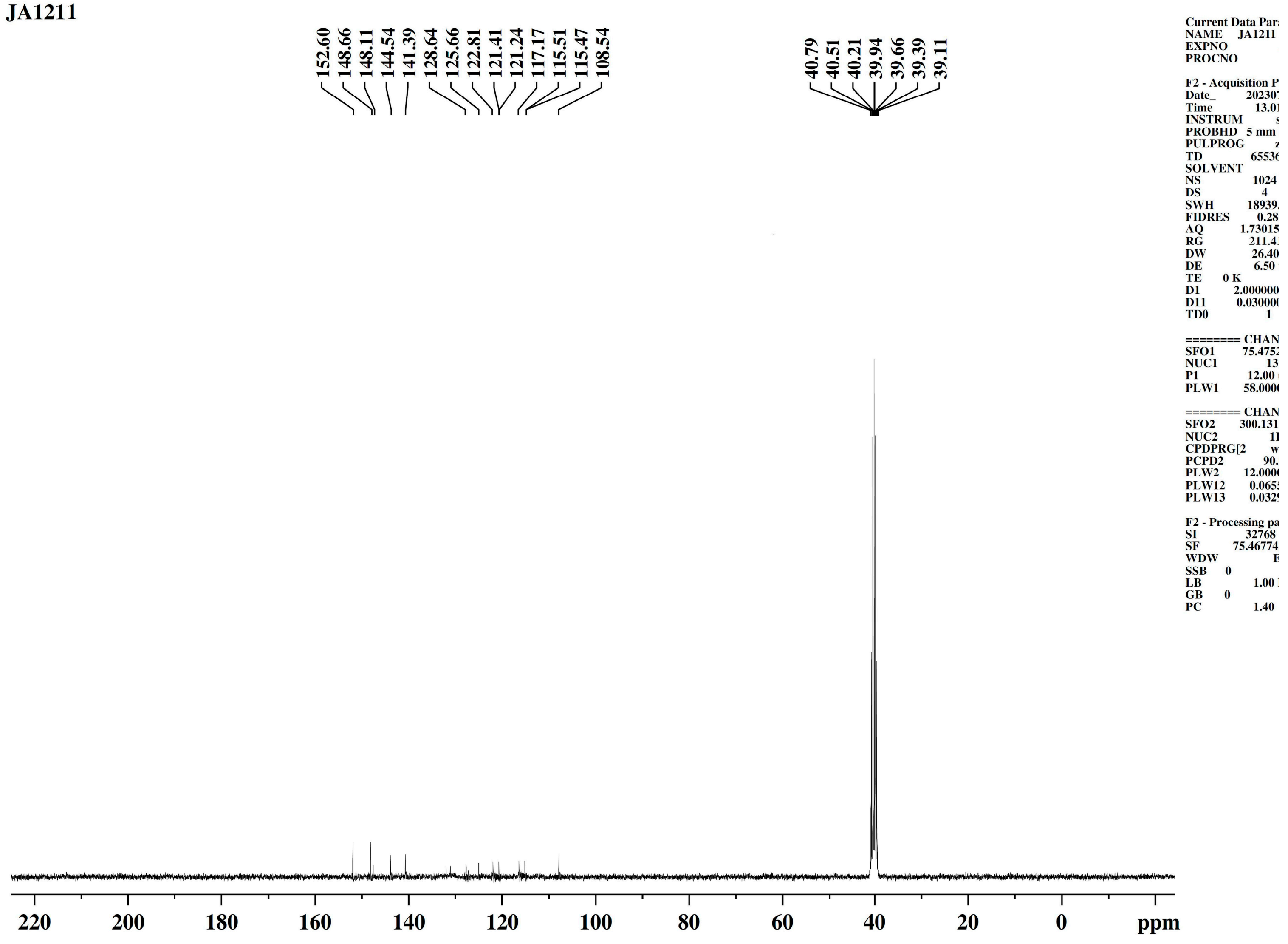

Figure S12. ^{13}C NMR spectra of 4-chloro-2-((5-(4-fluorophenyl)-1,3,4-oxadiazol-2-yl)amino)phenol (**6a**)

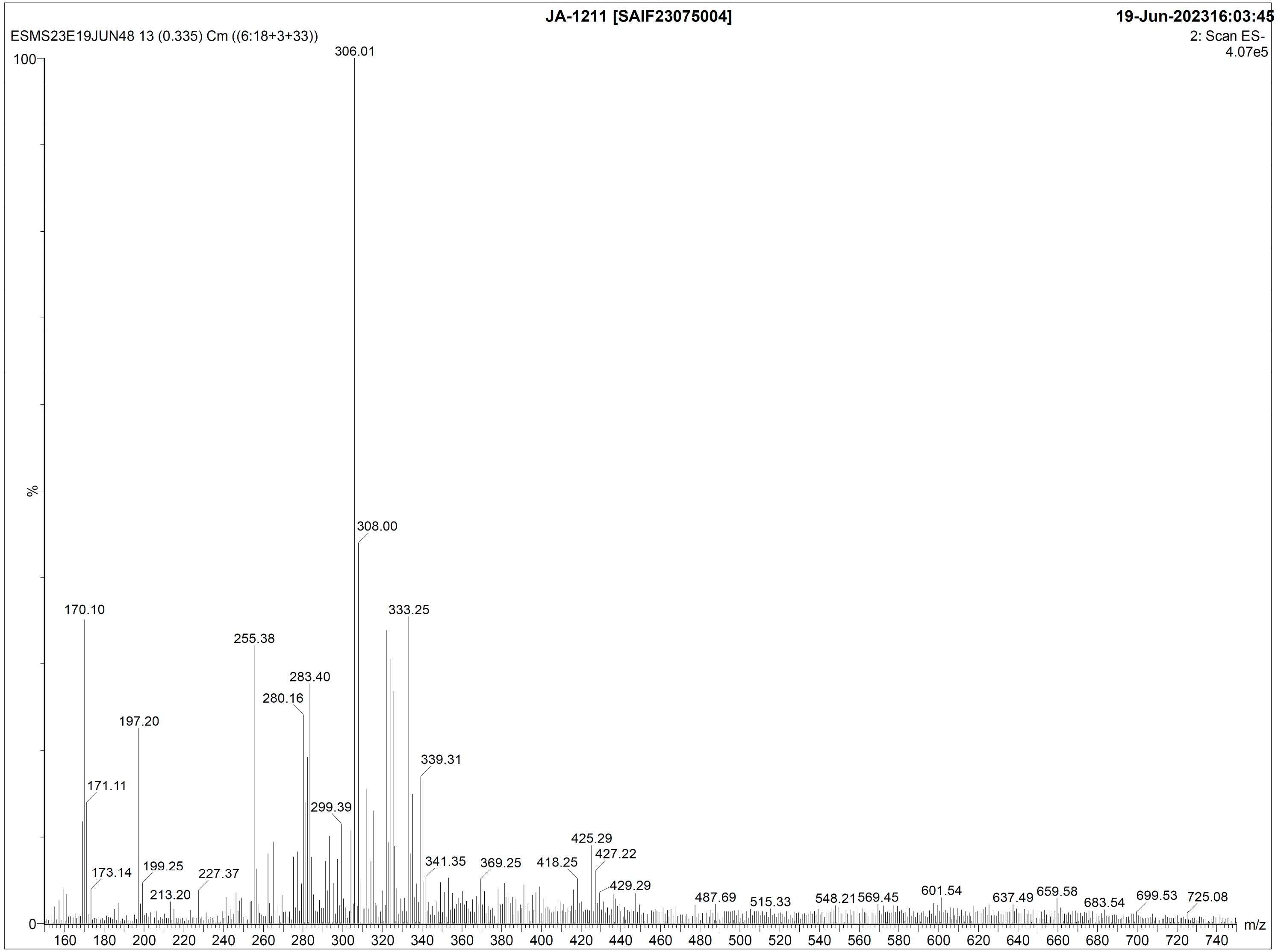

Figure S13. Mass spectra of 4-chloro-2-((5-(4-fluorophenyl)-1,3,4-oxadiazol-2-yl)amino)phenol (**6a**)

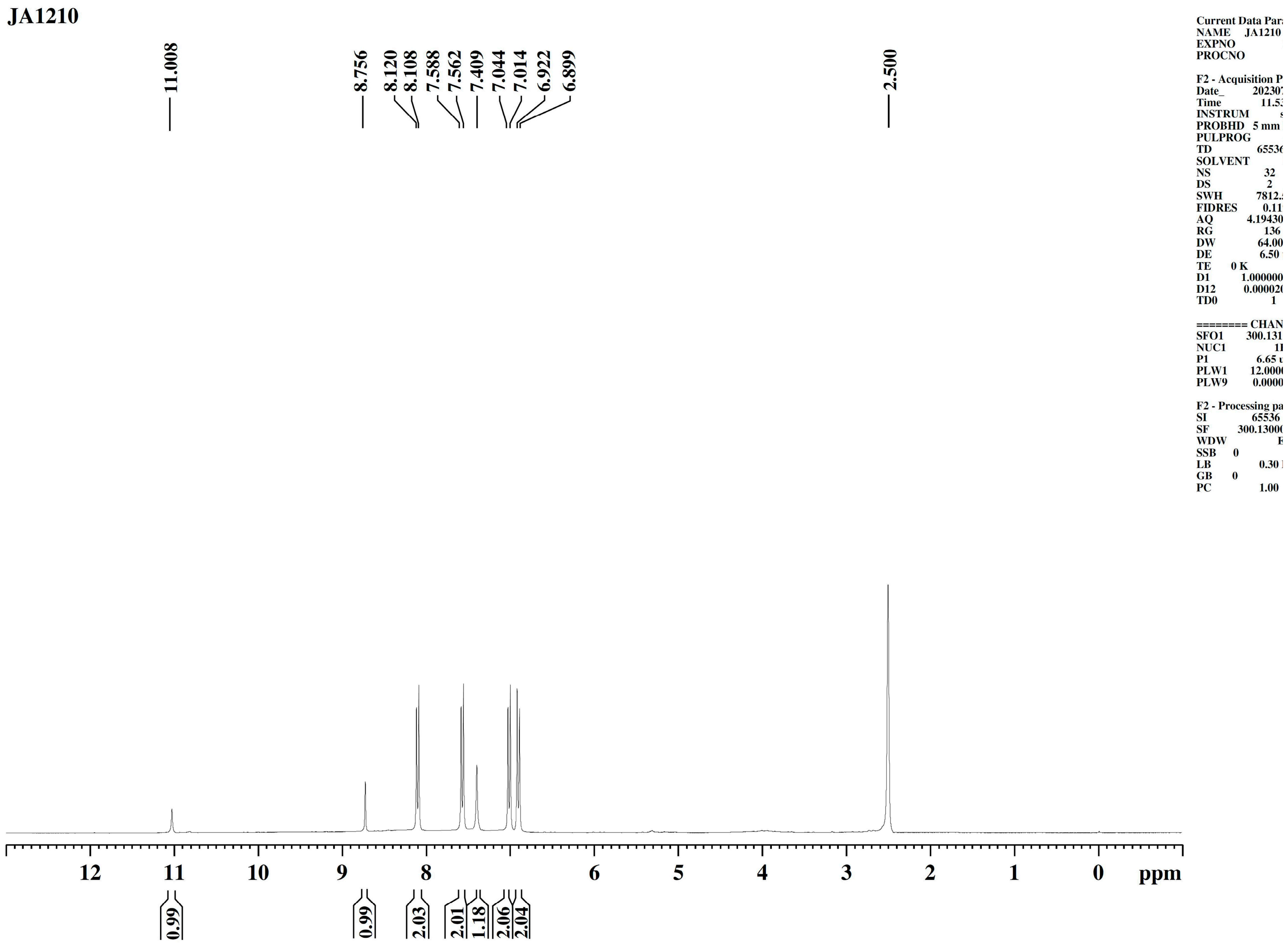

Figure S14. ^{1}H NMR spectra of 4-chloro-2-((5-(4-chlorophenyl)-1,3,4-oxadiazol-2-yl)amino)phenol (**6b**)

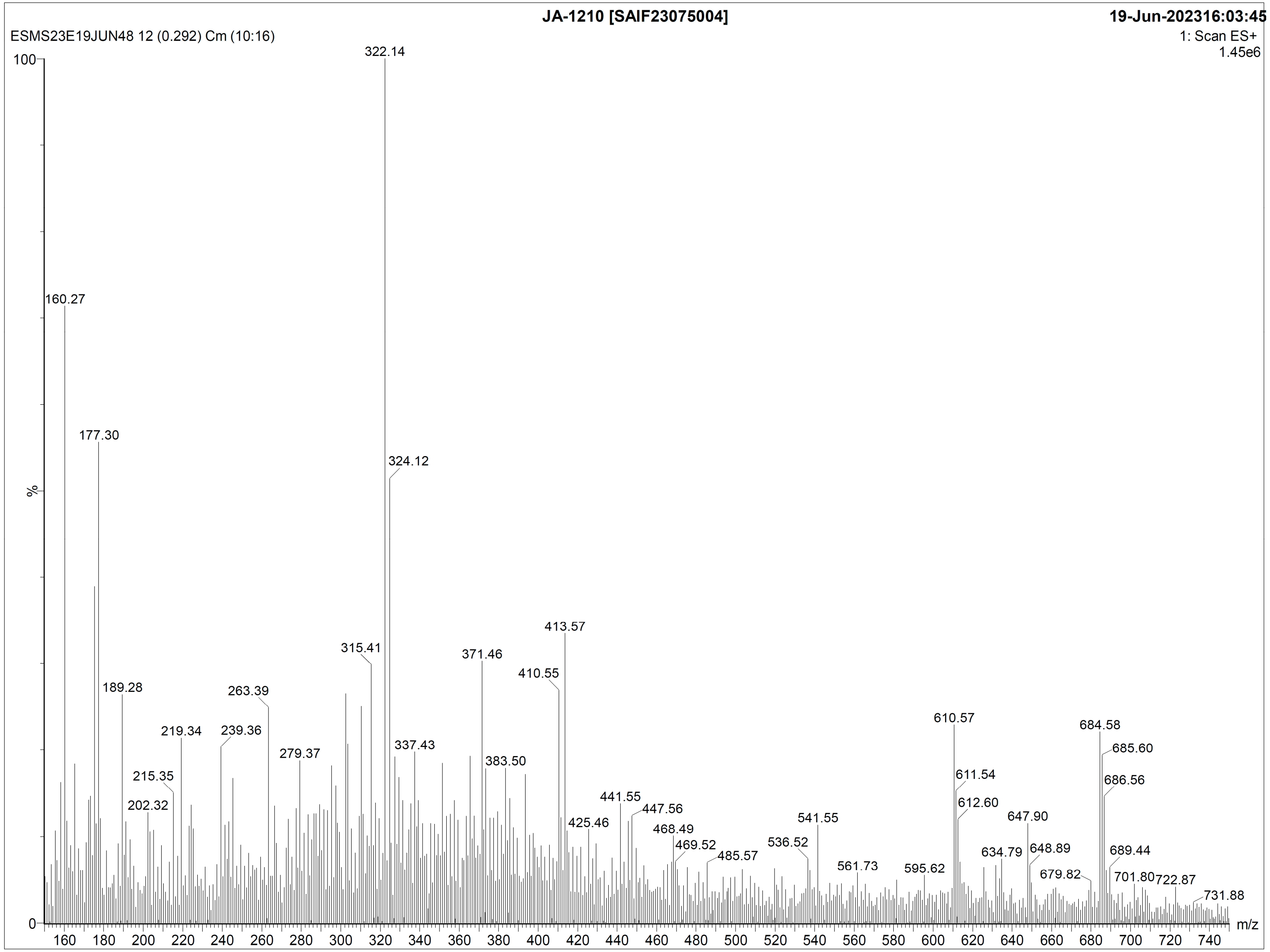

Figure S15. Mass spectra of 4-chloro-2-((5-(4-chlorophenyl)-1,3,4-oxadiazol-2-yl)amino)phenol (**6b**)

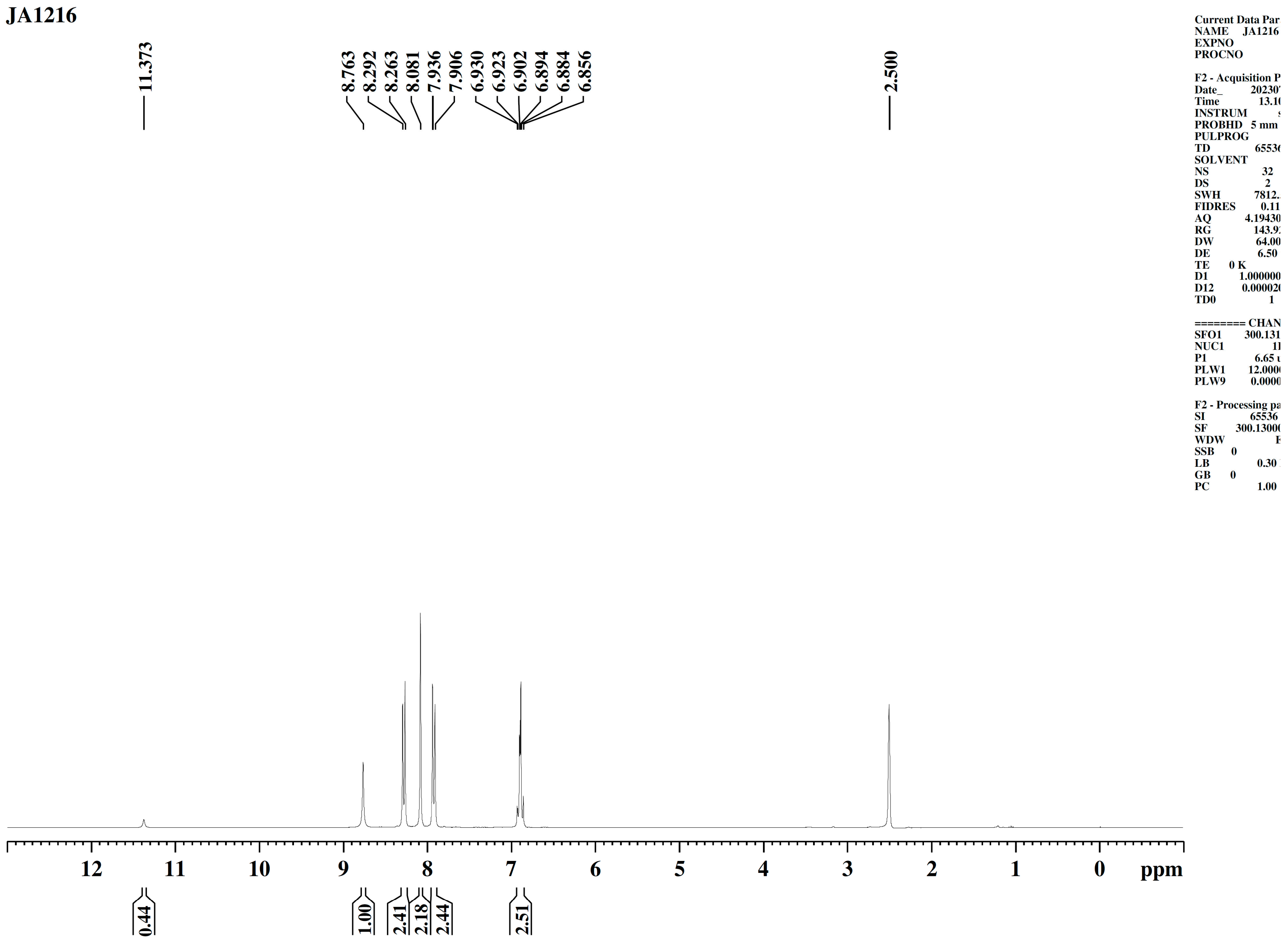

Figure S16. ^{1}H NMR spectra of 4-chloro-2-((5-(4-nitrophenyl)-1,3,4-oxadiazol-2-yl)amino)phenol (**6c**)

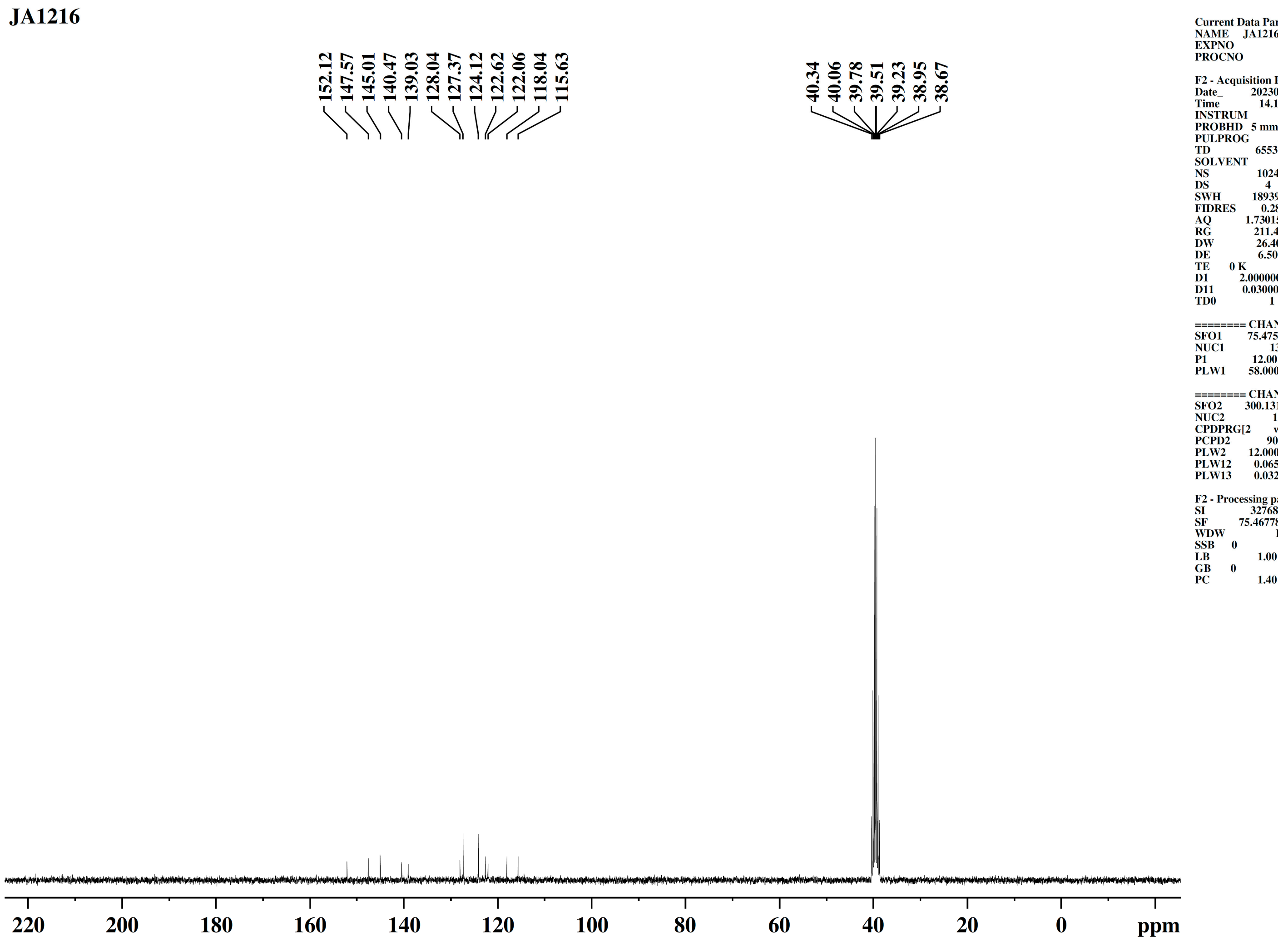

Figure S17. ^{13}C NMR spectra of 4-chloro-2-((5-(4-nitrophenyl)-1,3,4-oxadiazol-2-yl)amino)phenol (**6c**)

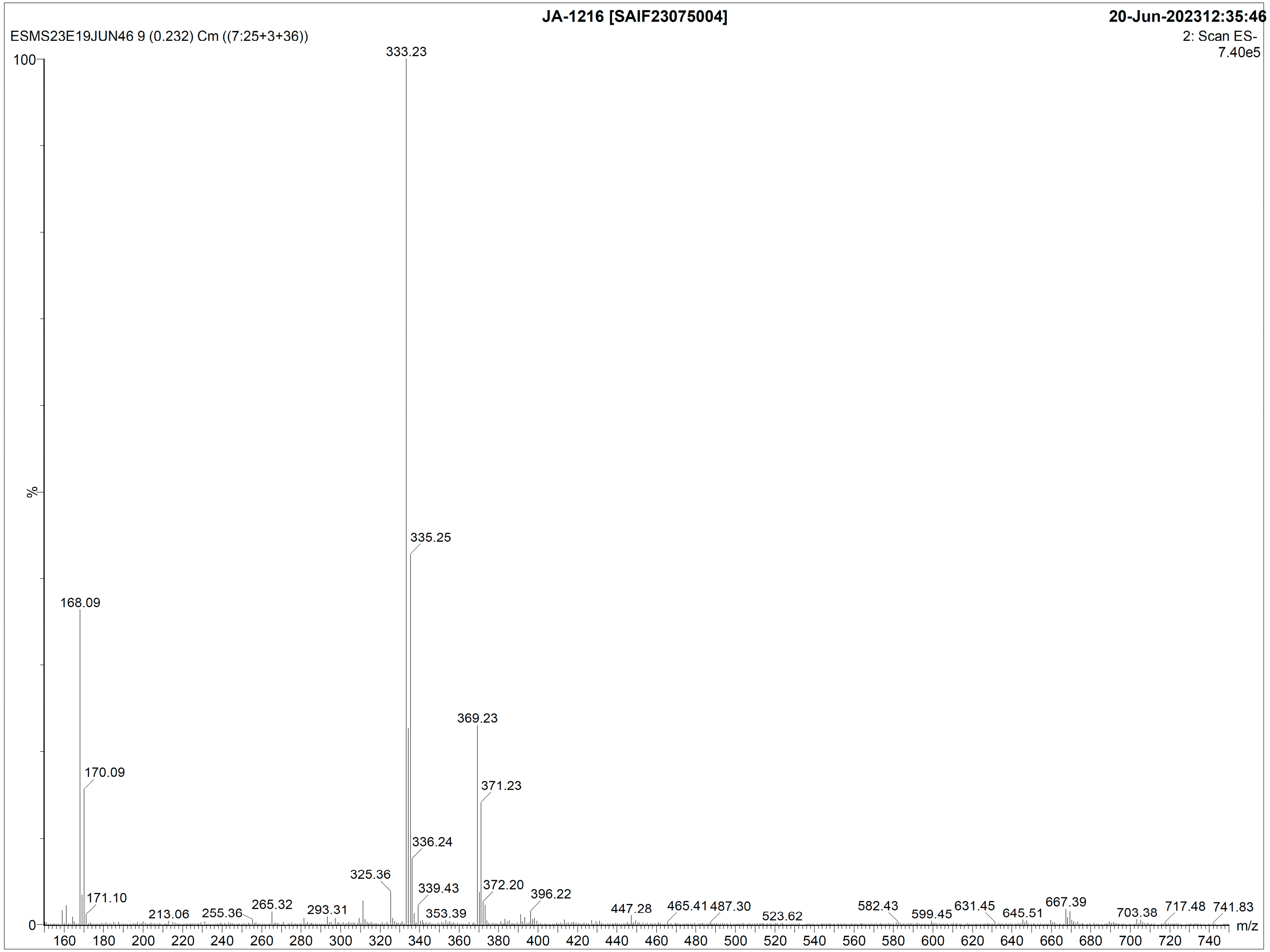

Figure S18. Mass spectra of 4-chloro-2-((5-(4-nitrophenyl)-1,3,4-oxadiazol-2-yl)amino)phenol (**6c**)

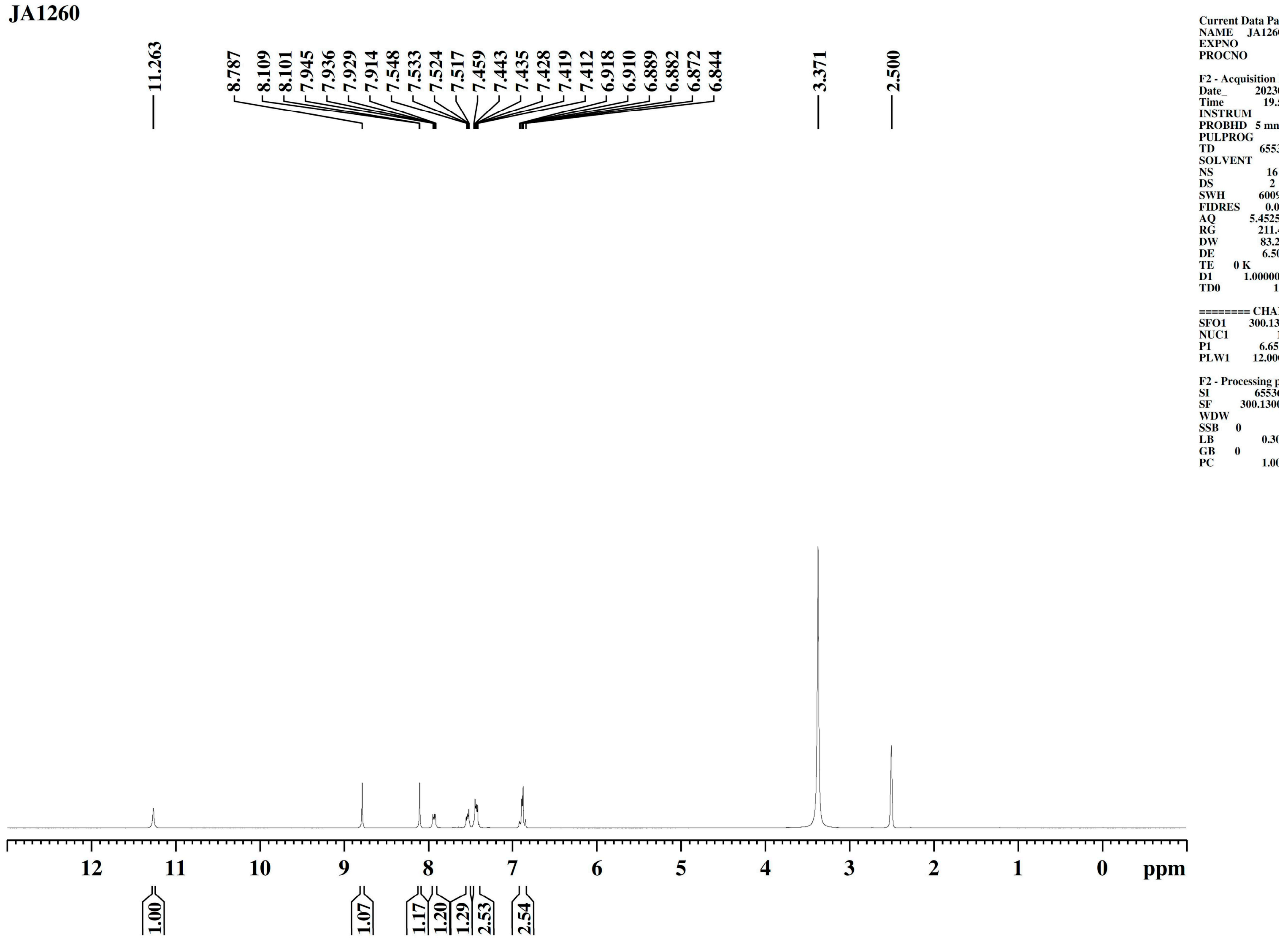

Figure S19. ^{1}H NMR spectra of 4-chloro-2-((5-(2-chlorophenyl)-1,3,4-oxadiazol-2-yl)amino)phenol (**6e**)

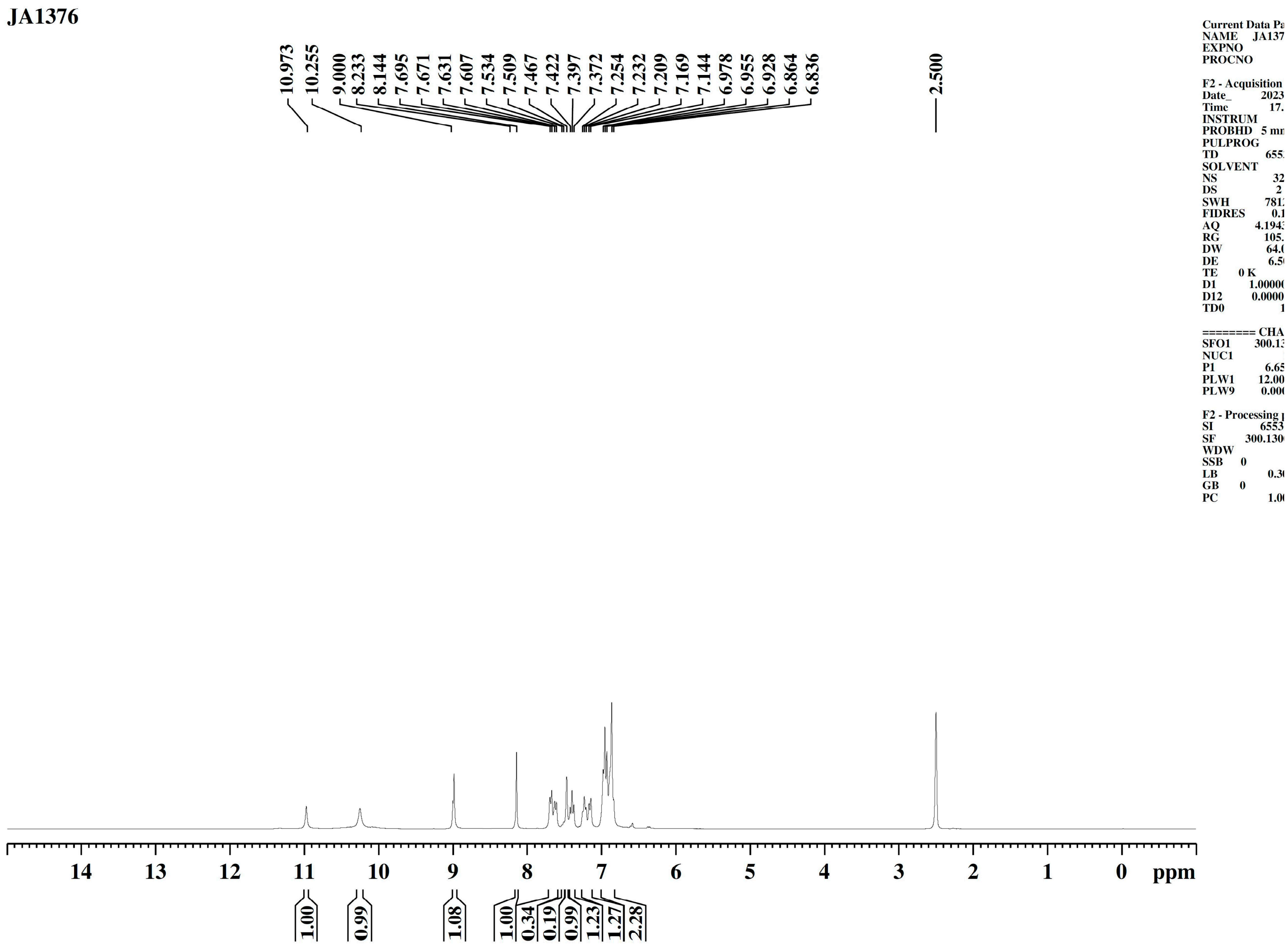

Figure S20. ^{1}H NMR spectra of 4-chloro-2-((5-(2-hydroxyphenyl)-1,3,4-oxadiazol-2-yl)amino)phenol (**6f**)

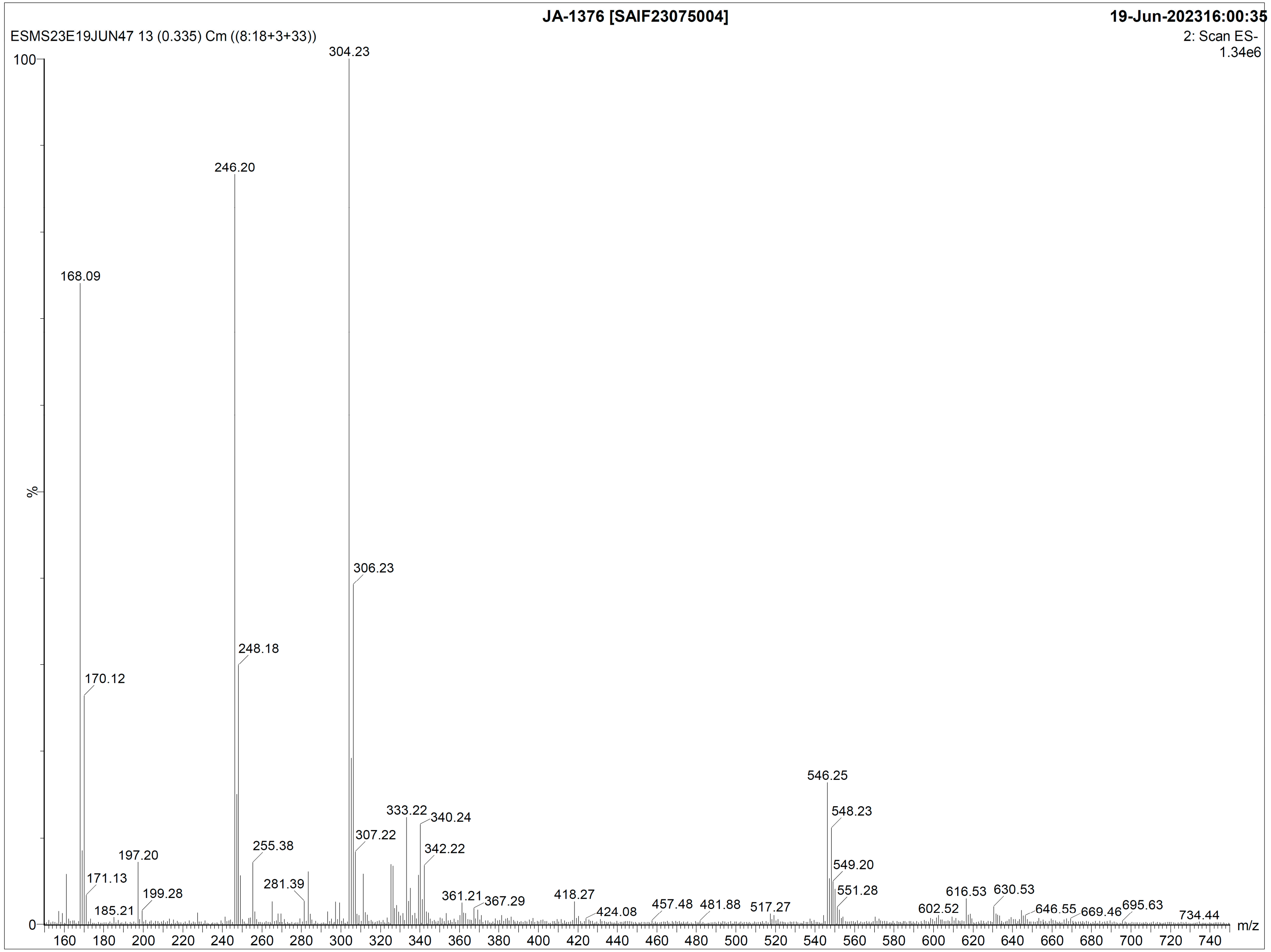

Figure S21. Mass spectra of 4-chloro-2-((5-(2-hydroxyphenyl)-1,3,4-oxadiazol-2-yl)amino)phenol (**6f**)

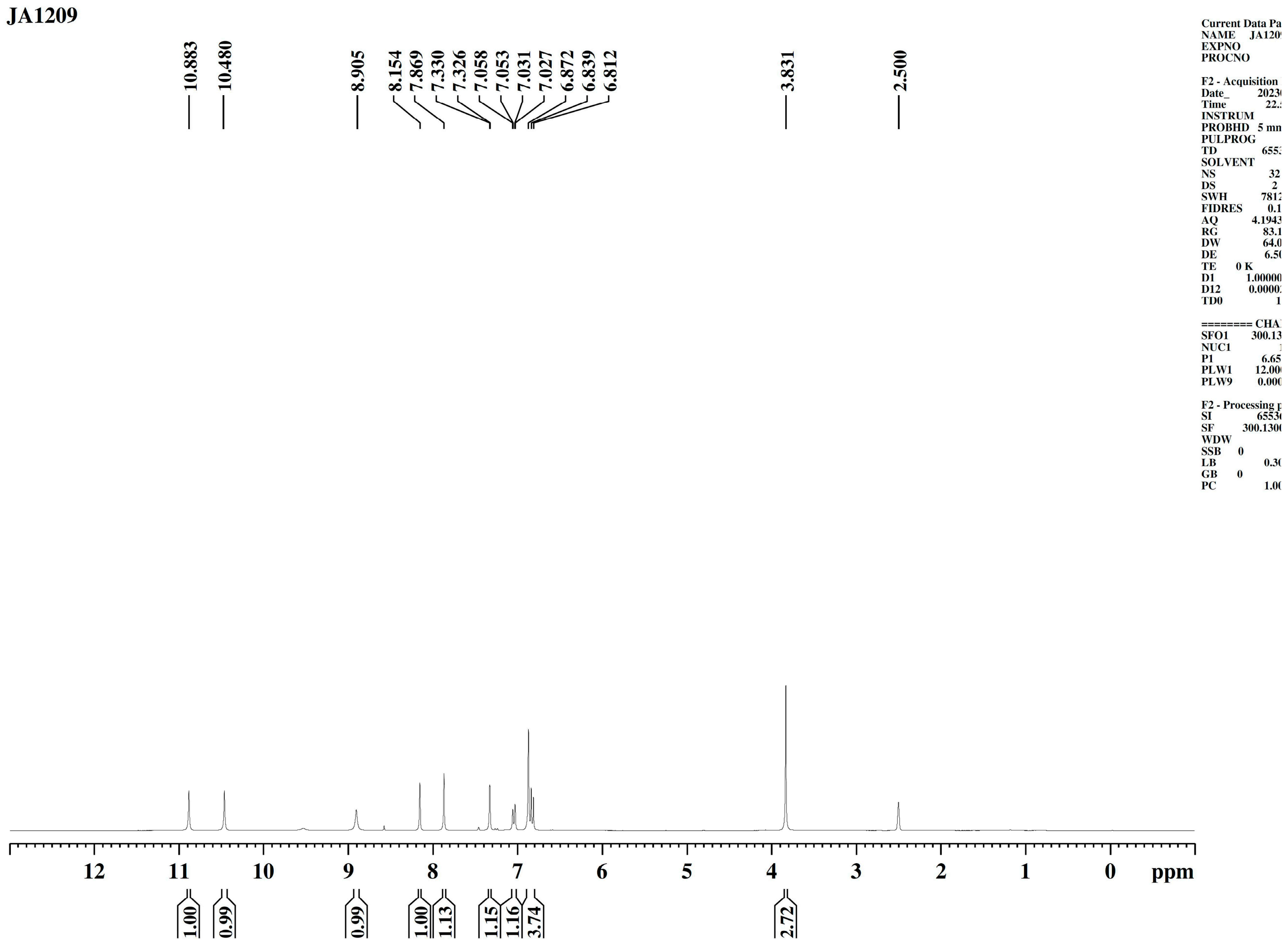

Figure S22. ¹H NMR spectra of 4-chloro-2-((5-(4-hydroxy-3-methoxyphenyl)-1,3,4-oxadiazol-2-yl)amino)phenol (**6g**)

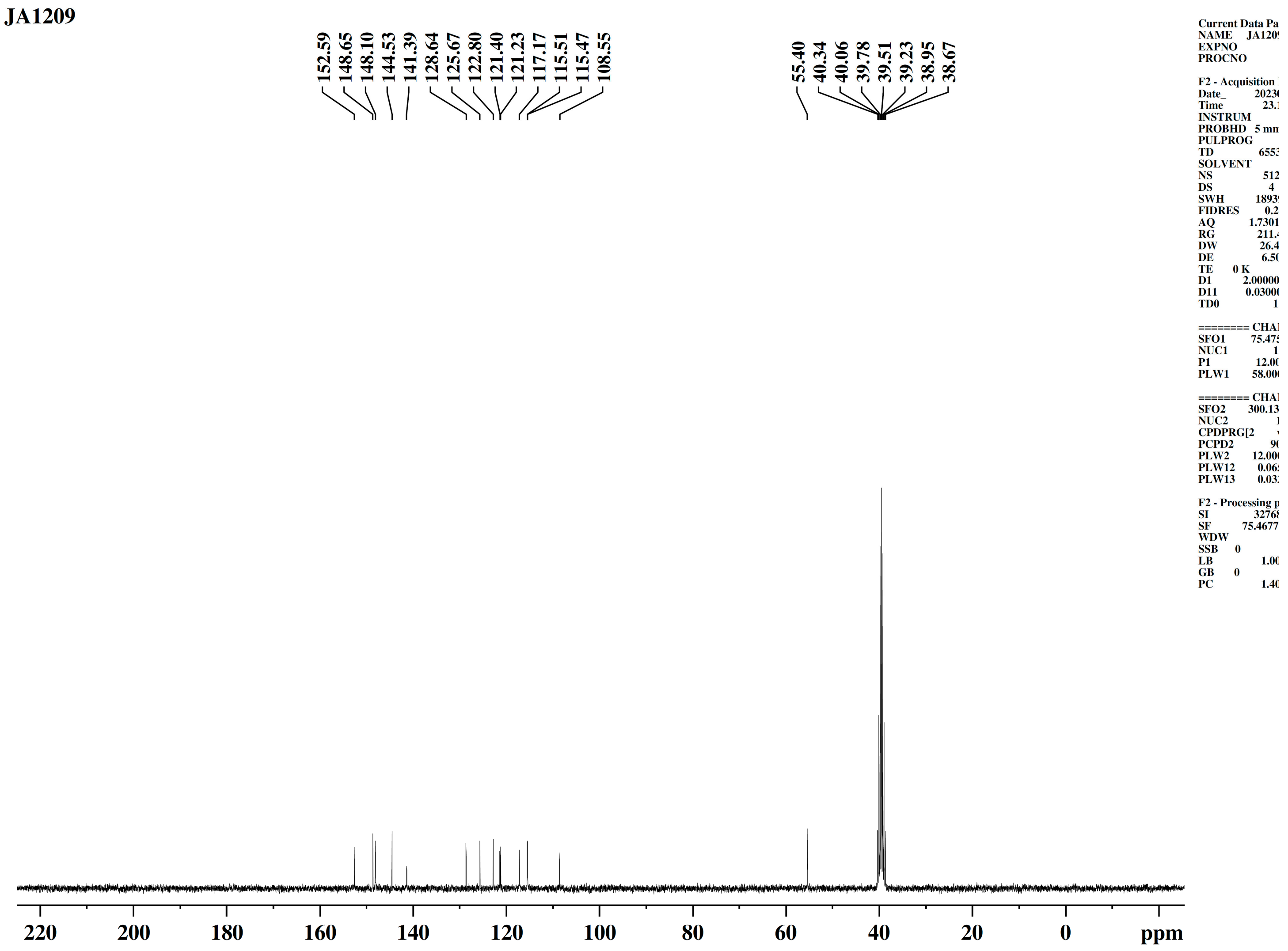

Figure S23. ^{13}C NMR spectra of 4-chloro-2-((5-(4-hydroxy-3-methoxyphenyl)-1,3,4-oxadiazol-2-yl)amino)phenol (**6g**)

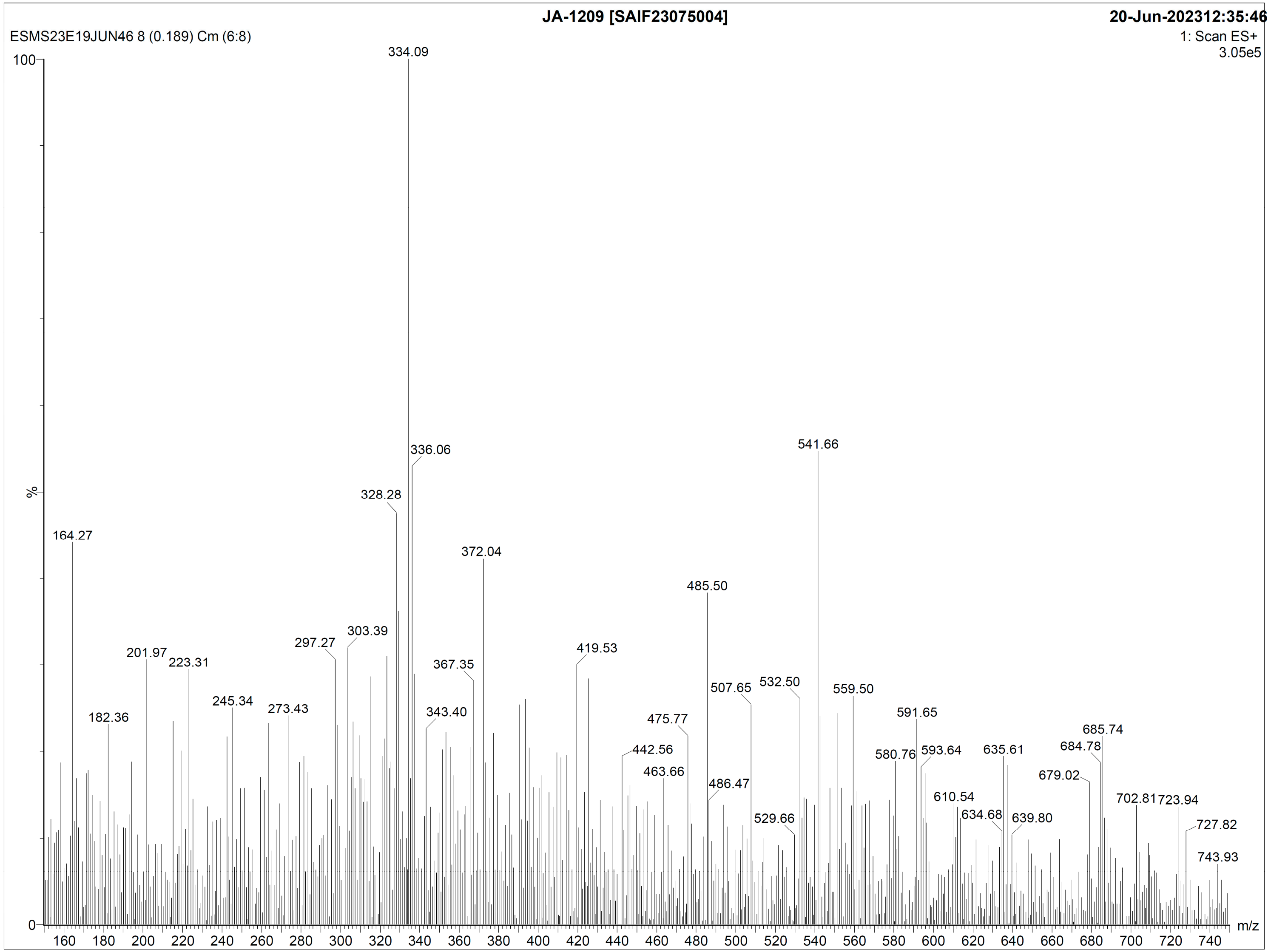

Figure S24. Mass spectra of 4-chloro-2-((5-(4-hydroxy-3-methoxyphenyl)-1,3,4-oxadiazol-2-yl)amino)phenol (**6g**)

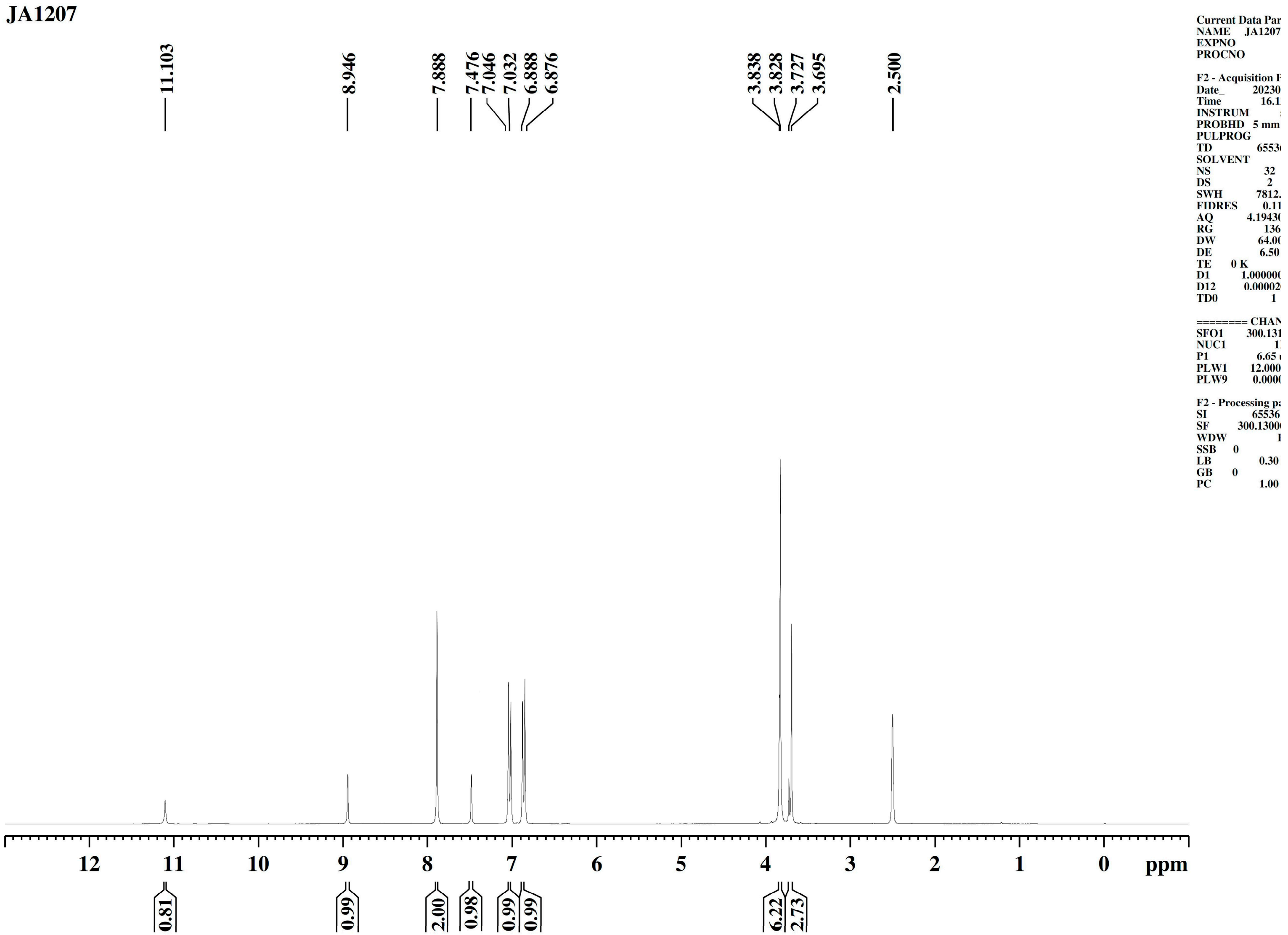

Figure S25. ^{1}H NMR spectra of 4-chloro-2-((5-(3,4,5-trimethoxyphenyl)-1,3,4-oxadiazol-2-yl)amino)phenol (**6h**)

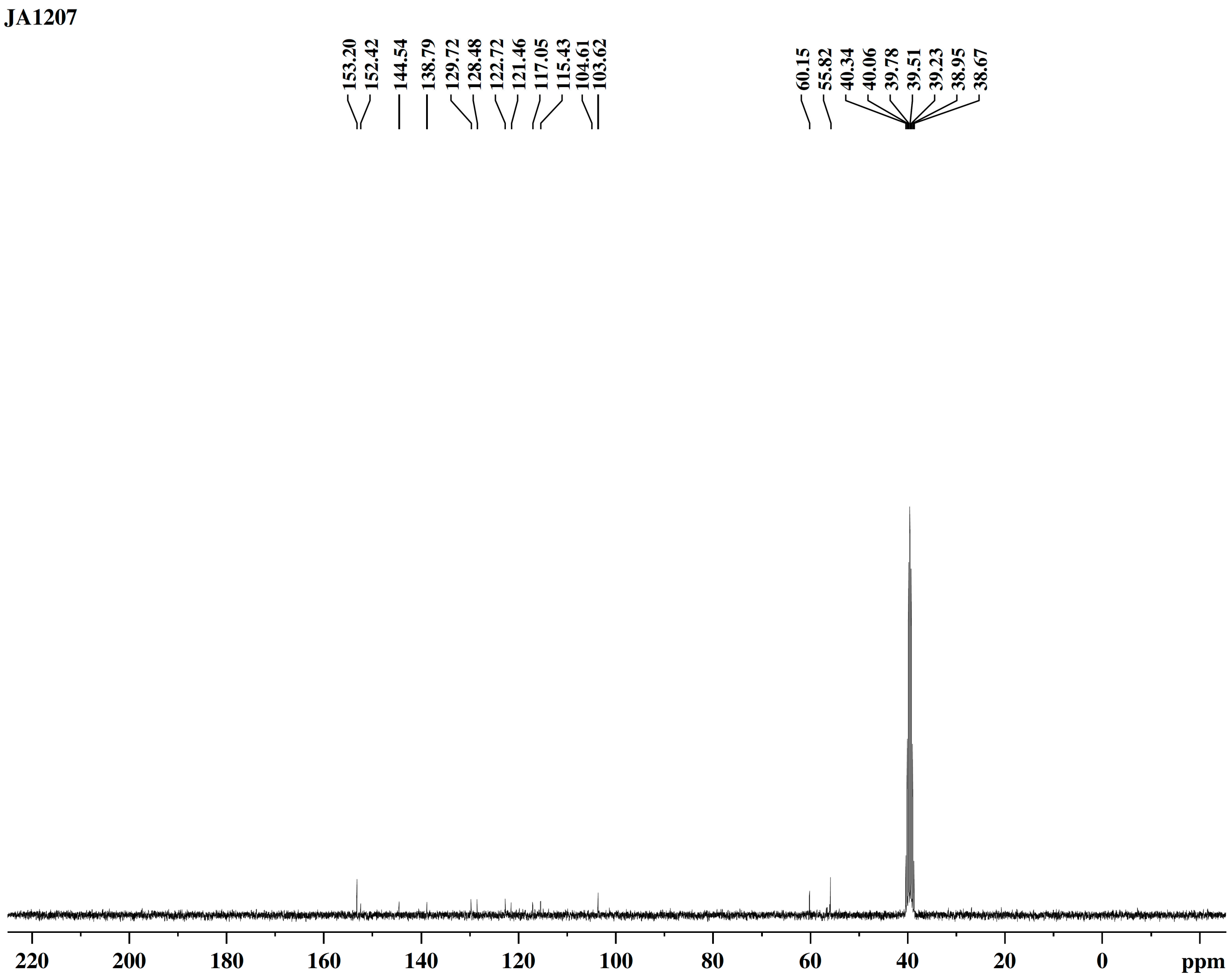

Current Data Par
NAME JA1207
EXPNO
PROCNO

F2 - Acquisition I
Date_ 20230
Time 16.4
INSTRUM
PROBHD 5 mm
PULPROG
TD 6553
SOLVENT
NS 512
DS 4
SWH 18939
FIDRES 0.28
AQ 1.7301
RG 211.4
DW 26.4
DE 6.50
TE 0 K
D1 2.00000
D11 0.03000
TD0 1

======== CHAN
SFO1 75.475
NUC1 13
P1 12.00
PLW1 58.000

======== CHAN
SFO2 300.131
NUC2 1
CPDPRG[2 w
PCPD2 90
PLW2 12.000
PLW12 0.065
PLW13 0.032

F2 - Processing p
SI 32768
SF 75.4677
WDW
SSB 0
LB 1.00
GB 0
PC 1.40

Figure S26. ^{13}C NMR spectra of 4-chloro-2-((5-(3,4,5-trimethoxyphenyl)-1,3,4-oxadiazol-2-yl)amino)phenol (**6h**)

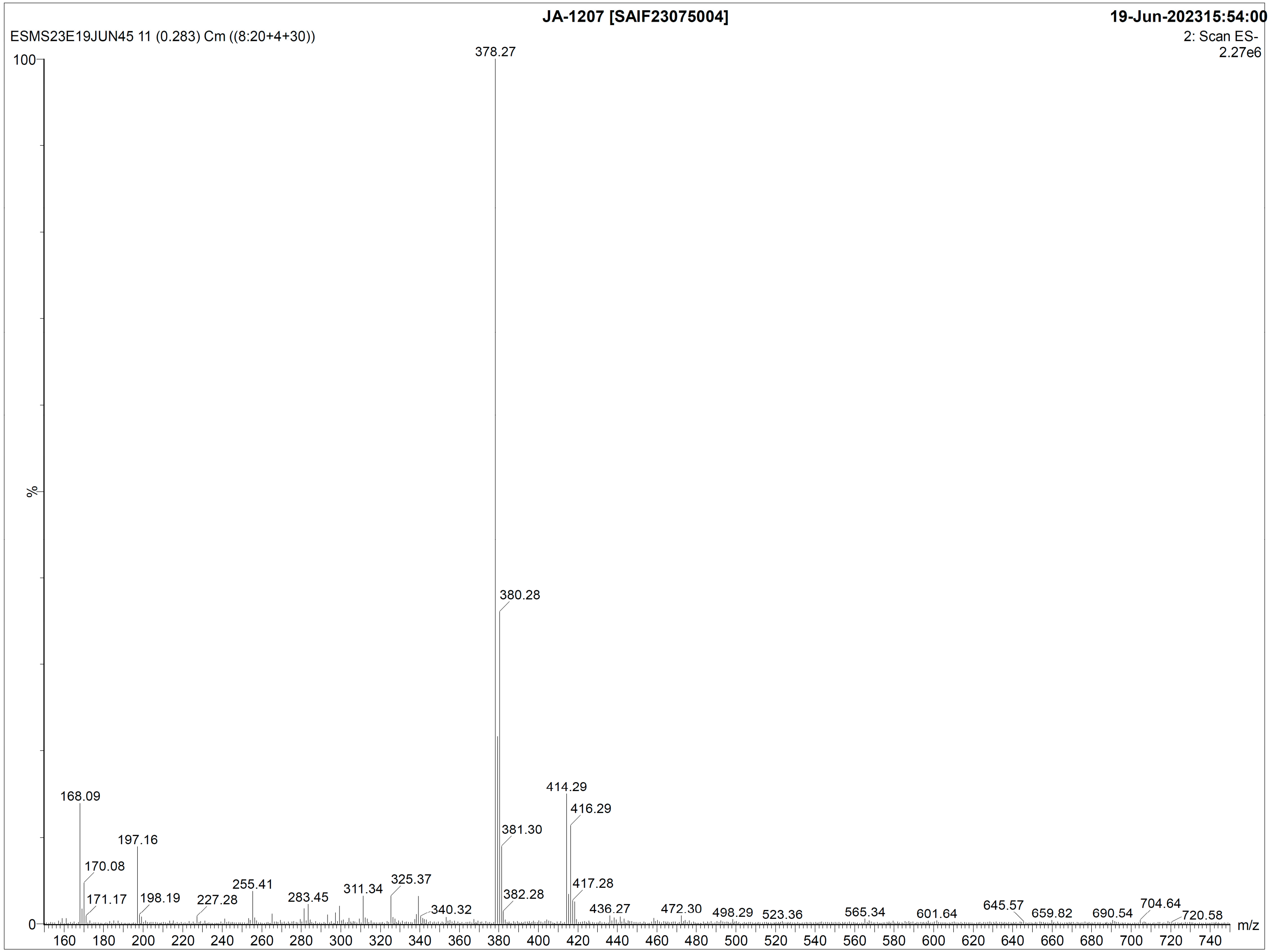

Figure S27. Mass spectra of 4-chloro-2-((5-(3,4,5-trimethoxyphenyl)-1,3,4-oxadiazol-2-yl)amino)phenol (**6h**)

HPLC GRAPH

Analyzer: Mr.Bhawani
Spl.Name: Unknown Sample-01
Wave.L:210 nm
Batch No:01
Status:
Flow:1.000 ml/min
Column: C18
Col.Temp:25
Date:2023-07-30 13:14:25

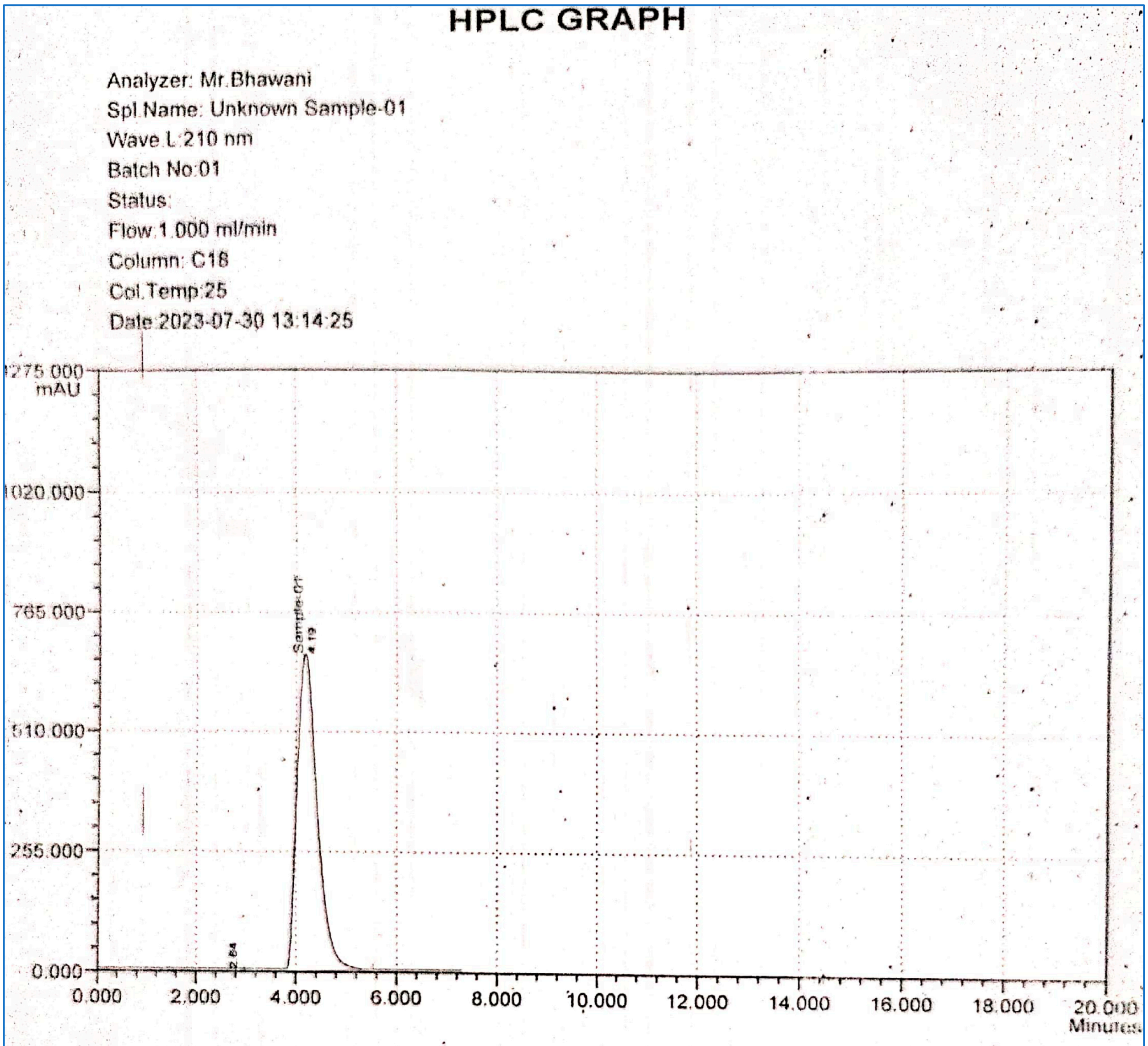

ID	Name	Retain.T	Height	Area	Conc	Tail.Factor	Theo.Plate
1		2.638	685	15281.3	0.808	1.69	279
2	Sample-01	4.186	67441	1877125.0	99.192	2.31	451
	Sum:		68126	1892406.3	100.0000		

Figure S28. HPLC chromatogram of 4-chloro-2-((5-(3,4,5-trimethoxyphenyl)-1,3,4-oxadiazol-2-yl)amino)phenol (**6h**)
